# Review of Watershed-Scale Water Quality and Nonpoint Source Pollution Models

**DOI:** 10.3390/geosciences10010025

**Published:** 2020-01-11

**Authors:** Lifeng Yuan, Tadesse Sinshaw, Kenneth J. Forshay

**Affiliations:** 1 National Research Council Resident Research Associate at the United States Environmental Protection Agency, Robert S. Kerr Environmental Research Center, 919 Kerr Research Drive, Ada, OK 74820, USA; 2 U.S. Environmental Protection Agency, Center for Environmental Solutions and Emergency Response, Robert S. Kerr Environmental Research Center, 919 Kerr Research Dr., Ada, OK 74820, USA

**Keywords:** watershed modeling, water quality, nonpoint source pollution, model selection, uncertainty analysis

## Abstract

Watershed-scale nonpoint source (NPS) pollution models have become important tools to understand, evaluate, and predict the negative impacts of NPS pollution on water quality. Today, there are many NPS models available for users. However, different types of models possess different form and structure as well as complexity of computation. It is difficult for users to select an appropriate model for a specific application without a clear understanding of the limitations or strengths for each model or tool. This review evaluates 14 more commonly used watershed-scale NPS pollution models to explain how and when the application of these different models are appropriate for a given effort. The models that are assessed have a wide range of capacities that include simple models used as rapid screening tools (e.g., Long-Term Hydrologic Impact Assessment (L-THIA) and Nonpoint Source Pollution and Erosion Comparison Tool (N-SPECT/OpenNSPECT)), medium-complexity models that require detail data input and limited calibration (e.g., Generalized Watershed Loading Function (GWLF), Loading Simulation Program C (LSPC), Source Loading and Management Model (SLAMM), and Watershed Analysis Risk Management Frame (WARMF)), complex models that provide sophisticated simulation for NPS pollution processes with intensive data and rigorous calibration (e.g., Agricultural Nonpoint Source pollution model (AGNPS/AnnAGNPS), Soil and Water Assessment Tool (SWAT), Stormwater Management Model (SWMM), and Hydrologic Simulation Program Fortran (HSPF)), and modeling systems that integrate various sub-models and tools, and contain the highest complexity to solve all phases of hydrologic, hydraulic, and chemical dynamic processes (e.g., Automated Geospatial Watershed Assessment Tool (AGWA), Better Assessment Science Integrating Point and Nonpoint Sources (BASINS) and Watershed Modeling System (WMS)). This assessment includes model intended use, components or capabilities, suitable land-use type, input parameter type, spatial and temporal scale, simulated pollutants, strengths and limitations, and software availability. Understanding the strengths and weaknesses of each watershed-scale NPS model will lead to better model selection for suitability and help to avoid misinterpretation or misapplication in practice. The article further explains the crucial criteria for model selection, including spatial and temporal considerations, calibration and validation, uncertainty analysis, and future research direction of NPS pollution models. The goal of this work is to provide accurate and concise insight for watershed managers and planners to select the best-suited model to reduce the harm of NPS pollution to watershed ecosystems.

## 1. Introduction

Nonpoint source (NPS) pollution is a pervasive source of water pollution in the world. In practice, a watershed is a fundamental unit of monitoring and management of NPS pollution. To better understand the complex mechanisms and processes of NPS pollution, various watershed-scale NPS models and tools were developed to understand NPS pollution, and to evaluate water quality. These NPS models and tools are widely used to identify critical source areas of pollutants [1–3], evaluate the effects of NPS pollution on the water environment [4,5], future scenarios evaluation of hydrology and water quality [6,7], assist in the planning and implementation of best management practices (BMPs) [8–10], support development of water quality criteria or standards [11,12], and provide informed decision support for policy-makers [13–15]. Models have become essential tools in the effort to reduce the adverse effects of NPS pollution. However, the current diversity of the NPS pollution models makes it difficult to select the most suitable model for a given NPS pollution issue.

The diversity of current models stems from a history of watershed management and planning demands. With the development of model theory, computer technology, and environmental legislation, numerous water quality and NPS pollution models were developed or integrated into watershed models (i.e., hydrology models or rainfall-runoff models) [16]. These models use the watershed as the fundamental spatial unit to simulate various hydrologic, hydraulic, soil erosion, sediment transport, and nutrient dispersion processes that accounts for surface water, groundwater, and their interaction as a whole system [17]. The Stanford Watershed Model, developed in 1959–1966, was the first computer model to conduct watershed hydrology analysis and modeling that subsequently evolved to the well-known Hydrologic Simulation Program Fortran (HSPF) in the 1970s [18]. Most of the current NPS pollution models such as HSPF [19], Soil and Water Assessment Tool (SWAT) [20], Agricultural Nonpoint Source pollution model (AGNPS) [21], Long-Term Hydrologic Impact Assessment (L-THIA) [22], Generalized Watershed Loading Function (GWLF) [23], Source Loading and Management Model (SLAMM) [24], Stormwater Management Model (SWMM) [25], and Better Assessment Science Integrating Point and Nonpoint Sources (BASINS) [26] were developed during the 1970s–1990s. In this period, NPS models were individually developed to solve a specific watershed issue. After the 1990s, the comprehensive application of models, databases, visualization representation of results gradually became a widespread solution to support decisions related to watershed-scale NPS pollution. Large improvements in data availability and data resolution (e.g., GIS, remote sensing, and electronic sensor technology) [27–29] and the integration between NPS pollution research and other data-driven methods such as artificial intelligence (e.g., artificial neural network) [30] and machine learning (e.g., support vector machine) [31,32] have become common for NPS pollution model development.

Because many NPS models are available for users, a clear understanding of the function and structure of the NPS pollution model is essential for its appropriate application to avoid its misinterpretation and misapplication in practice [33]. Some review articles or reports that assist in model identification for water quality and NPS pollution problems have been published in the scientific literature in the last two decades. Deliman, et al. [34] summarized eleven watershed water quality models including hydrology, sediment and pollution components, and intended use of each model, and broke these models into two groups (urban and nonurban) by land-use types. Urban models include Distributed Routing, Rainfall, Runoff Model (DR3M), Storage, Treatment, Overflow Runoff Model (STORM), and SWMM. Nonurban models are Chemical, Runoff, and Erosion from Agricultural Management Systems (CREAMS)/Groundwater Loading Effects of Agricultural Management System (GLEAMS), Erosion/Productivity Impact Calculator (EPIC), Simulator for Water Resources in Rural Basins (SWRRB), Water Erosion Prediction Project (WEPP), Pesticide Root Zone Management (PRZM), AGNPS, HSPF, and SWAT. Upon reviewing models, they recommended two comprehensive models HSPF and SWAT to users. Borah and Bera [33,35] reviewed 11 watershed-scale models that focus on hydrologic and NPS pollution prediction, and categorized them into three groups by time scale of simulation: AGNPS, Areal Nonpoint Source Watershed Environment Response Simulation (ANSWERS), Dynamic Watershed Simulation Model (DWSM), and KINematic runoff and EROSion model (KINEROS) are used in simulating single storm event and estimating watershed management practices; Annualized Agricultural NonPoint Source (AnnAGNPS) pollution model, ANSWERS-Continuous, HSPF, and SWAT are suitable to analyze long-term hydrologic response to agriculture management practices; CASCade of planes in 2-Dimensions (CASC2D), MIKE SHE, and Precipitation-Runoff Modeling System (PRMS) can conduct both long-term and single event simulations. They also discussed the mathematical bases of these watershed-scale models. After analyzing the applications of each model, they proposed that SWAT, HSPF, and DWSM are three promising models in predominantly agricultural watersheds, mixed agricultural and urban watersheds, and a single storm event, respectively. Fu, et al. [36] explored the publication records of 42 water quality and soil erosion models based on Scopus literature database, and discussed in detail the five most commonly used models (SWAT, HSPF, eWater Source, Integrated Catchment Model (INCA) [37], Spatially Referenced Regression on Watershed Attributes (SPARROW)) [38]. They categorized and compared these five models from the viewpoint of model use, model development, and model performance, involving physical process representation, spatial and temporal scale, data requirement, calibration, validation, and uncertainty tools, as well as that the future challenges in the field of water quality modeling particularly related to large data and accurate predictions.

**Figure 1.**
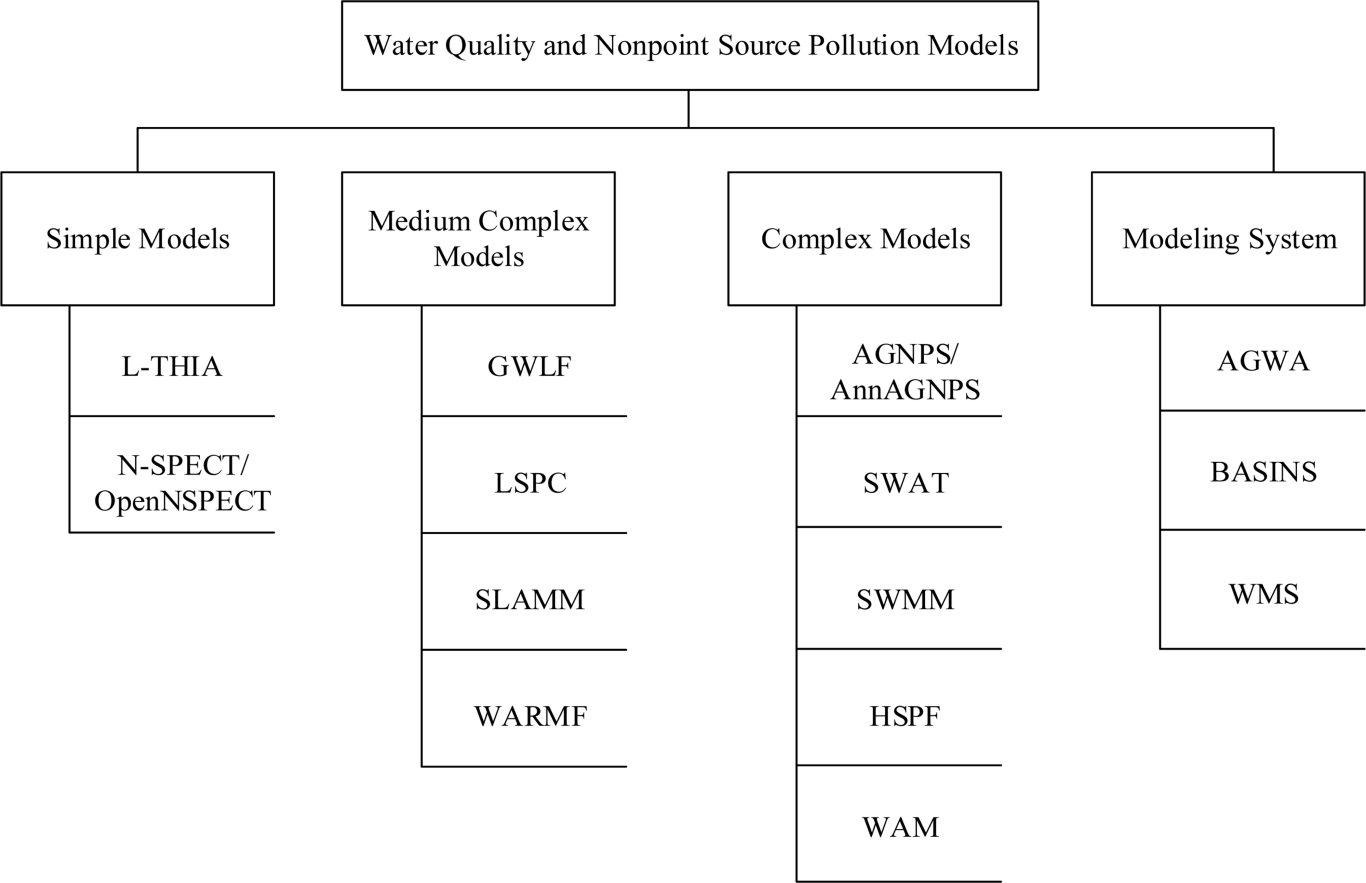
Classification of water quality and NPS pollution models.

In spite of the aforementioned review articles, there is limited guidance on how to select an appropriate model for the purpose of watershed management and planning. In this article, 14 commonly used watershed-scale NPS pollution models that predict flow, sediment and nutrient concentrations or loads, and estimate watershed water quality were evaluated. These models include: AGNPS, BASINS, GWLF, HSPF, L-THIA, SLAMM, SWAT, SWMM, Automated Geospatial Watershed Assessment Tool (AGWA) [39], Loading Simulation Program C (LSPC) [40], Nonpoint Source Pollution and Erosion Comparison Tool (N-SPECT) [41], Watershed Analysis Risk Management Frame (WARMF) [42], Watershed Assessment Model (WAM) [43], and Watershed Modeling System (WMS) [44]. These models were evaluated and compiled with their key attributes including the primary intended use, model components, suitable land-use type, input parameter types, spatial and temporal scales, pollutants, strength and weakness, software developers or access, and availability. The above models were categorized into four groups: simple models, medium complexity models, complex nodels, and modeling system (Figure 1). Details about each group are explained in Section 2. Most of these discussed NPS pollution models are no charge for public use. As this review was limited to a subset of well-known watershed-scale NPS pollution models that are in use, many other useful field-scale NPS pollution models and receiving water bodies or in-stream water quality models such as QUAL series [45] and Water Quality Analysis Simulation Program (WASP) [46], and other metamodels such as Smart prediction of the Impact of Management strategies (nutrient and water) on Phosphorus losses by LEaching (SIMPLE) [47] and others are not included in this review. In this work we develop a simple classification structure of model complexity and provided common examples of watershed-based water quality models in these complexity categories to provide insight for users and practitioners.

We attempted to not only reflect the main features of each watershed model, but also discussed current challenges in model selection such as spatial and temporal scale, calibration and validation, and uncertainty analysis. We also discussed the future potential direction of NPS pollution model research. This review will help modelers understand how these tools should be applied in practice and be useful for practitioners of watershed management and planning to make an informed decision while choosing an appropriate model for their application related to water quality and NPS pollution.

## 2. Watershed-Scale Nonpoint Source Pollution Model Evaluation

Watershed-scale NPS models can be classified according to a variety of criteria that include the methods used to quantify hydrologic processes (e.g., empirical, conceptual, or physically-based), spatial variability of input parameters (e.g., lumped, semi-distributed, or distributed), spatial and temporal scales (e.g., small, field, or watershed; event-driven or long-term simulation), or the uncertainty of model output (e.g., deterministic or stochastic) [48]. To simplify model selection, we categorized models into four groups: simple models, medium complexity models, complex models, and modeling systems. This classification considers the intrinsic structure and form of the tools and models as well as the data and calibration requirements necessary to apply the model. This provides a framework that also helps the end user understand the appropriate application and requirements for implementation of the model tool. Table 1 summarized the main characteristics of different NPS pollution model types.

### 2.1. Simple Models

Simple models have minimal data requirements that includes land-use, soil, precipitation, and digital elevation model (DEM) (optional for some specific areas). This type of model is built based on an empirical or statistical relationship between pollution loads or concentrations, land use, rainfall, and runoff volume. These models typically use the Soil Conservation Service-Curve Number (SCS-CN) method [49] to calculate overland runoff and employed the export coefficient or the Event Mean Concentration (EMC) method [50] to calculate pollution loading. Simple models are often used as quick screening tools to obtain the gross pollution loads at the outlet of a watershed or to evaluate long-term areal average pollution loads for a moderate or large size watershed [51]. Although the simple models described here can provide good generalized information with little calibration, some validation of models is preferred. Simple models do not consider the spatial route of flow, sediment and pollution transport, nor are pollutant fate and transport mechanism in water bodies included. Thus, these models possess limited capacity to simulate detailed hydrological and physicochemical processes. These tools alone may not be sufficient to assist in water pollution control decisions that require insight on opportunities or locations to implement prevention or regulatory measures. These tools are also limited to a small number of simulated pollutants. Simple models can be powerful tools for generalized understanding of the pollutant loads or concentrations but neglect details in physical processes of NPS pollution.

#### 2.1.1. L-THIA

L-THIA (Long-Term Hydrologic Impact Assessment), developed by Purdue University, is designed to estimate the long-term effects of land-use change in urban or suburban areas on surface runoff, groundwater recharge, and NPS pollution [22]. L-THIA includes three versions: L-THIA basic model, a spreadsheet version of L-THIA runs on the internet [52]; ArcL-THIA, an ArcGIS-based extension tool [53]; L-THIA low impact development (LID) estimates the impacts of land-use changes and LID practices on urban runoff and water quality [10].

L-THIA was designed to quickly evaluate average annual runoff volumes and quantify NPS pollutant loads drained into water bodies in urban and suburban areas. L-THIA does not require intensive data input and can provide ‘what-if’ alternative future scenario analyses. The L-THIA model consists of hydrology and water quality components. L-THIA uses the runoff curve number (CN) method to determine direct runoff and employs the EMC method to calculate annual pollutant loads. Lim, et al. [22] improved the model by using a single rainfall event to estimate average yearly NPS pollution for 15 pollutants. Contaminants incorporated by L-THIA include nitrogen, phosphorus, suspended sediment loads, bacteria, and metals [54].

L-THIA was successfully applied to evaluate NPS pollution in different places such as United States [55], South Korea [22], and China [56]. Zhang, et al. [56] used L-THIA to evaluate the spatial distribution of NPS pollution in Qingdao (10,654 km^2^) China, and verified that L-THIA is a reliable model to provide informed decision for land use management and planning. You, et al. [57] simulated and validated nitrogen (N) and phosphorus (P) loads across different land-use using L-THIA model in the Sihu basin (11,547.5km^2^) in China and found that the model performed well for estimating the average loads of N and P. Liu, et al. [58] used L-THIA-LID as an optimization tool to estimate the impact of land use and climate change on hydrology and water quality with future scenario analysis in Trail Creek Watershed (153.2 km^2^) in northwest Indiana, United States.

Overall, L-THIA is a quick screening tool for NPS pollution and water quality evaluation in urbanized areas. Users with limited knowledge of hydrology and the environment can also utilize the L-THIA model. The data needed for model running are easily attainable, especially in the United States. The application of L-THIA needs no or only limited calibration when it is applied to watersheds across the Midwest U.S. [59]. However, L-THIA can only reflect the average annual runoff volume and pollutant loads, and ignores the spatial route of flow, sediment and pollutants. The assumption of this model does not include pollutant fate and transport in receiving water bodies. Since the EMC method itself considers pollution concentration as a constant over time, L-THIA cannot reflect a change of management practice (e.g., fertilizer application) on land, nor express the dynamic relationship between seasonal or varied flow and concentration [36]. Validation of the L-THIA model remains a significant challenge without site specific data where the model is applied, especially in an application of a large watersheds that may have heterogeneity in land use, land cover, precipitation, soil, or locations that are not preloaded with topographic data.

#### 2.1.2. N-SPECT/OpenNSPECT

N-SPECT (Nonpoint Source Pollution and Erosion Comparison Tool), developed by National Oceanic and Atmospheric Administration (NOAA), allows managers to quickly examine relationships between potential water quality of water bodies, NPS pollution, and soil erosion in nearshore areas [41]. N-SPECT works as a plug-in extension of ArcGIS, and the latest version of N-SPECT is compatible with ArcGIS 9.3. However, users must have a license of ArcGIS and its spatial analysis tool before running this model. To expand access to users without an ArcGIS commercial license, the NOAA Coastal Services Center developed OpenNSPECT in 2011, which is a free and open-source version of N-SPECT. OpenNSPECT is a plug-in tool of open-source MapWindow GIS and has the same theoretical foundation as N-SPECT [60]. The software, user’s manual and documentation of OpenNSPECT are available at [61].

N-SPECT/OpenNSPECT estimates runoff volume, sediment yield, and pollutant loads/concentrations, identifies soil erosion susceptible areas, and estimates the impact of land-use changes on water quality [41]. The model can operate at an annual or event time step in a medium-to-large near-shore watershed, and support ‘what-if’ scenario analysis under different land use management practices. In N-SPECT/OpenNSPECT, the runoff CN method is used to estimate surface runoff; the EMC method is applied to calculate pollutant concentration; the Revised Universal Soil Loss Equation (RUSLE) and Modified Universal Soil Loss Equation (MUSLE) are employed to estimate erosion rate and sediment loads; the Sediment Delivery Ratio (SDR) method is used to evaluate sediment yield. Finally, the model generates an overall water quality rating and allocates this rating to river networks by comparing the simulated total pollutant concentrations to local water quality standards [41]. Evaluated pollutants include total nitrogen (TP), total phosphorus (TP), total suspended solids (TSS), zinc, and lead. Input data sets include DEM, land use, soil, slope, precipitation, rainfall and runoff erosivity (R-factor), local pollutant coefficients, and water quality standards. The model outputs have runoff volume and depth, accumulated pollutant loads and concentrations, soil erosion, and total sediment yield [41,62].

N-SPECT/OpenNSPECT was applied to understand and evaluate the effects of land use management practices on water quality, especially for nearshore ecosystem health. Maina, et al. [63] applied the N-SPECT to estimate annual sediment load per unit area in two catchments of Madagascar island in Australia to examine the relationship between coral reefs and environmental change in coastal watersheds. Butler, et al. [64] used N-SPECT model to calculate runoff volume and changes in the annual delivery of total N for each scenario analysis in Tully Murray catchment in Australia. Tulloch, et al. [65] employed OpenNSPECT model to simulate runoff and sediment discharge from New Ireland watershed (7404 km^2^) while evaluating the impacts of the oil palm industry in the nearshore ecosystem health in Papua New Guinea.

N-SPECT/OpenNSPECT is a light-level screening tool for estimating water quality, soil erosion, sediment yield, and NPS pollution at a nearshore watershed. The model does not require intensive data input and is a simple model structure based on GIS raster calculation. Although the model manual claims that the model can be applied to any watershed, recent applications demonstrated that the model is often applied to ecosystem health assessment in coastal areas [63,66]. N-SPECT/OpenNSPECT can simulate the distinct contributions of sediment and pollutants from upstream areas. However, the model is not a sophisticated tool for watershed water quality and NPS pollution assessment. The model does not account for stormwater drainage, stream diversions, snowmelt, sediment redeposition and the dynamic processes of runoff, sediment, and pollutant transport [67].

### 2.2. Medium Complexity Models

Unlike the simple models discussed above, medium complexity models generally require relatively detailed data inputs such as topography, land use, soil, weather, and water quality. The type of model is typically used as a compromise between simple models and complex models. Medium complexity models not only account for the fundamental water and sediment movement processes, but also combine the empirical relationships to express nutrient loads. Meanwhile, it avoids applying a complicated physically-based watershed model that requires intensive data. The calculation of surface runoff is based on a water balance principle [68]. The temporal step of some models estimates a daily value of runoff, sediment, and nutrient loads. Although medium complexity models do not require rigorous calibration, model validation is also preferable. Most of these models can assist in the development of water quality criteria and are easy to operate compared to complex models. However, these models have inherently restricted simulation capability for in-stream fate and transport of pollutants, and the number of simulated pollutants is limited as well.

#### 2.2.1. GWLF

GWLF (Generalized Watershed Loading Function), initially developed by Haith and Shoemaker [23], is used to estimate monthly flow, sediment, and nutrient loading, and assists TMDLs development from a medium-size urban or agricultural watershed with various land-use combinations [69]. The latest version of GWLF is integrated into the Mapshed model that currently maintained by Pennsylvania State University with online access at [70].

GWLF combines distributed/lumped parameters and estimates long-term continuous flow, sediment, and nutrient loads from the land surface into streams based on daily weather data input [1]. GWLF can simulate dissolved and solid-phase nutrient loads in streamflow and considers different pollution sources such as surface runoff, groundwater, and septic systems. In agricultural land, GWLF uses the CN method to calculate runoff and obtain dissolved nutrient loads by multiplying runoff volume by dissolved nutrient concentration from each land use type. GWLF computes solid-phase nutrient loads by multiplying monthly sediment yields by average sediment nutrient concentrations. Soil erosion can be obtained by applying a modification of the Universal Soil and Erosion Equation (USLE). Sediment yield is generated from soil erosion, runoff transport capacity, and sediment delivery ratio (SDR) [69]. In urban land, GWLF calculates runoff by SWMM [71] and STORM model [72]. Pollutant loads are regarded as entirely solid-phase, and calculated by exponential accumulation and washoff functions [69]. In groundwater, pollutant loads are spatially-lumped for a watershed and calculated as the product of subsurface flow and a watershed average nutrient concentration [73]. The inputs of GWLF include precipitation, temperature, runoff sources and transport, and chemical parameters on a daily time step. The model outputs include monthly flow, monthly soil erosion and sediment yield, monthly TN and TP loads in flow, monthly groundwater discharge to flow, annual erosion from each land-use type, and yearly TN and TP loads from each land use type [73].

GWLF was applied to assess the environmental impact of soil erosion and NPS pollution [27,74], estimate streamflow, sediment and nutrient loads [75], as well as simulate daily flow [68]. Niraula, et al. [1] applied GWLF and SWAT to identify the critical source areas of NPS pollution in the Saugahatchee Creek watershed (570 km^2^) in east-central Alabama. Their findings showed that both models can accurately evaluate streamflow, but SWAT had a better performance in terms of predicting sediment yield, TN, and TP. Qi, et al. [76] also compared the performances of GWLF and SWAT in simulating monthly streamflow, sediment yield, and total N loads in the Tunxin catchment (2674 km^2^) and the Hanjiaying basin (6736 km^2^) of China. Their results support the fact that GWLF is easy to use since it requires fewer data to conduct simulations compared to the data needed for SWAT. Similarly, Gene [11] also demonstrated that GWLF is easy to use and is less complicated than SWAT and HSPF.

GWLF can be applied to an ungauged watershed, and it needs no calibration or only minimal calibration. If calibration is not conducted, all calibration parameters should be estimated with a similar method in the application of GWLF to a watershed [11]. However, GWLF only first identifies nutrient loads from different land-use types, then applies these results to the entire basin. The model needs distributed parameters as input for surface pollutant simulation but does not account for a spatial structure or channel routing of the flow component. In groundwater modeling, GWLF uses lumped parameters with a linear reservoir model that ignores the spatial variability of physical and chemical transport processes [76].

#### 2.2.2. LSPC

LSPC (Loading Simulation Program C^++^), developed by Tetra Tech Inc. with funding from U.S. EPA, is a C^++^ version of the HSPF model that can simulate hydrology and water quality [77]. The current version of LSPC is 5.0 that was released in 2015. Users can download LSPC 5.0 installable files and its user manual from the link [78].

Since LSPC rewrites the code of selected HSPF components in C^++^, the model has the same core algorithm of HSPF [40]. The main new features of LSPC include a modular structure, an input file organization form, model segmentation, meteorological linkage, data input/output, routines and other capabilities that are not included in HSPC. LSPC can simulate flow, soil erosion and sediment transport, general water quality from both upland contributing areas and receiving water bodies, as well as modules for stream transport, total maximum daily loads (TMDL) calculation, and source allocations in an urban or agricultural watershed [79]. The model is driven by hourly precipitation and can model hourly surface runoff and subsurface flows. The spatial scale of LSPC application is applicable to a small or large size watershed composed of over 1000 sub-watersheds. A Microsoft Access database is used to manage data and weather files in ASCII format. The primary components of LSPC include hydrology, snow, temperature, irrigation, sediment, as well as water quality sub models like the general quality (GQUAL) and the reach quality (RQUAL) [77]. The simulated pollutants include sediment, nutrients, metals, dissolved oxygen (DO), biochemical oxygen demand (BOD), plankton, and other contaminants from pervious and impervious lands. The input of LSPC includes DEM, soil, land use, and meteorological data. The model produces a time series of nutrient loading, hydrographs, and impacts of predetermined Best Management Practices (BMPs) [79].

LSPC is an efficient and flexible watershed hydrology and water quality model [80]. Shen and Zhao [81] employed LSPC to model surface runoff and subsurface flow while estimating bacteria nonpoint source loading in Sandy Bottom Branch watershed (6.9 km^2^) in Virginia. They found that LSPC can reasonably simulate streamflow over a 10-year period. Huang and Xiang [82] applied LSPC to investigate point source and NPS pollution of the Panjiakou Reservoir basin (42,443 km^2^) in north China. They indicated the developed model based on LSPC had sufficient accuracy in representing the hydrological characteristics of the watershed.

LSPC was designed to facilitate data management, organization, and modeling for a large complex watershed such as a municipal boundary (e.g., state or province) scale. LSPC has no inherent limits on the size and spatial-temporal resolution of input data, and the model uses a Microsoft Access database to manage model configuration and parameter files [79]. LSPC incorporates point source and NPS pollution, and also can be applied to water quality criteria like TMDL development. The output from LSPC can also be easily linked with other in-stream water quality models such as Environmental Fluid Dynamics Code (EFDC), WASP, CE-QUAL-W2, and System for Urban Stormwater Treatment and Analysis Integration (SUSTAIN). However, LSPC does not allow multiple sub-basins to connect to a single reach, nor deal with complex groundwater routing to simulate interactive process between surface and subsurface water [79].

#### 2.2.3. SLAMM

SLAMM (Source Loading and Management Model), initially developed by Robert Pitt and John Voorhees, is a pollutant source area identification and management planning model for an urban stormwater runoff [24]. The most recent version of SLAMM is a Windows-based WinSLAMM 10.4.1 that was released in 2019, jointly developed by The U.S. Geological Survey (USGS) and the Wisconsin Department of Natural Resources. WinSLAMM is not a public domain software, and it is currently maintained by PV & Associates, LLC. WinSLAMM can be available from [83].

SLAMM/WinSLAMM is an event-based continuous urban stormwater quality model and planning tool that can predict runoff discharges and pollutant loads for each source area within each land use type [84]. The model exams the relationships between source areas of urban pollutants and stormwater runoff quality, including evaluating contributions of source areas, estimating stormwater outfall discharge, particulate washoff, stormwater controls practices, and predicting pollutant concentration and loads [85]. The types of urban land-use in SLAMM involve residential, institutional, commercial, industrial, open space, and freeways and corresponding 14 source areas for each land use. The SLAMM calculates pollutant concentration and loads by using the discharge volume and suspended solids concentrations at the outfall. The simulated pollutants include particulate solids, P, TKN, COD, metals, and fecal coliform bacteria. The input data include storm, pollutant probability distribution, runoff coefficient, particulate solids concentration, street delivery on different land-use types, particle size distribution on each source area and flow peak of average flow ratio. The output includes runoff and flow summary, outfall and source area totals, source areas by land use, and outfall for each rain [86]. The improvement of WinSLAMM includes tracking pollution particle size distribution through stream network and report pollutant reduction from each land-use type and control device.

SLAMM/WinSLAMM has shown its reliability in predicting the impacts of stormwater control practices on flows and pollutant loads. Hurley and Forman [14] used WinSLAMM to evaluate the potential reductions of phosphorus loading to the Charles River in the Allston Campus Institutional Site (0.75 km^2^) and Zakim Industrial Area (0.81 km^2^) in Boston. Selbig, et al. [87] employed WinSLAMM to analyze the impact and the spatial distribution of particles in stormwater on the required size of stormwater control measures intended to meet pollutant reduction target.

SLAMM/WinSLAMM primarily depends on field observations rather than pure theoretical estimates that have not been widely verified in practice. The model was built based on the theory of small storm hydrology [88] with the concept that stormwater contamination is caused by frequent, small or moderate rain events [85]. The model considers different stormwater controls for a long series of rains. The model highlights water quality simulation, rather than only treating it as a part of hydrology or physcial hydraulics [14]. However, the current versions of WinSLAMM do not consider snowmelt, baseflow conditions, or account for in-stream processes that can remove or transform pollutants. Also, the model cannot simulate mass erosion from urban construction sites. Additionally, WinSLAMM is not a public domain software and its help documentation is simple.

#### 2.2.4. WARMF

WARMF (Watershed Analysis Risk Management Framework), mainly developed by System Water Resources, Inc., is a comprehensive decision support system for watershed management and analysis, and can support water quality criteria development [89]. However, WARMF is not public domain software. Users can obtain the software from [89].

WARMF can predict short or long-term physical, biochemical processes in any river basin, and includes vertical and lateral flow, groundwater, sediment loads, the fate and transport of nitrogen, phosphorus, metals and pesticides, and supports watershed management criteria development such as TMDL calculations, point/nonpoint source pollutants analysis, and watershed water quality planning [90,91]. WARMF can continuously simulate hydrologic processes with a daily time step. WARMF is composed of five interlinked modules, that include the engineering module, consensus module, data module, manager module, knowledge module, and TMDL module. The engineering module is used to estimate hydrology and water quality. The consensus module is employed to evaluate management practices and conduct scenario analysis. The data module is used to edit database files, and represents outcome with graphs and spreadsheets. The manager module is designed for real-time watershed management. The knowledge module collects various laws/regulations and case studies regarding the watershed. The TMDL module instructs the user to calculate a single pollutant from a point source or nonpoint source for a watershed to meet designated criteria [42]. The simulated pollutants include inputs of acid mine drainage, inputs from septic systems, bacteria, DO, mercury loading and transport, sediment, periphyton in rivers, and algae in stratified reservoirs. The input of WARMF includes DEM, land use, soil, meteorology, air quality, point source discharge, monitored streamflow, and water quality. The final products have a TMDL implementation plan or watershed management plan to support control of point and NPS pollution.

Geza, et al. [92] used WARMF to a mountain watershed (126 km^2^) and evaluates predictive uncertainty by using a calibration and uncertainty analysis algorithm. Dayyani, et al. [91] developed DRAIN-WARMF to improve the deficiency of WARMF, which was used to simulate subsurface flow and the nitrogen fate and transport of a small agricultural watershed (24.3 km^2^) in eastern Canada.

WARMF is a based on the physical movement of water, sediments, and nutrients in a watershed. The tool is suitable for locations like acid mine drainage, mercury pollution, and on-site wastewater systems. WARMF is stand-alone software that possesses a GIS interface, so users do not need to procure additional GIS licenses to drive the model. However, WARMF does not account for a tile drainage system, deep groundwater aquifers, and groundwater quality [93].

### 2.3. Complex Models

Complex models can simulate NPS pollution processes based on intrinsic physical processes. These models generally integrate hydrology, erosion and sediment processes, and chemical pollutant fate and transport. The data required for these models are commonly large. Complex models consider the estimation of runoff, soil erosion, sediment and pollutant loading based on theoretical consideration of mass, momentum, and energy [94]. Complex models can not only address a wide range of watershed-scale hydrology and water quality issues, that include short- and long-term simulations of runoff, sediment, and pollutant loads, but also supply different algorithm options for various physical processes to more accurately describe the processes mathematically. These models must be calibrated and validated carefully before applying them in management decisions because poor data input or inappropriate application of algorithms can lead to large errors [95]. These models have detailed documentation that often require extensive training and/or experience to apply correctly. These complex models require intensive parameters inputs to drive the model calculations, which are often unavailable in some areas. Careful parameter sensitivity analyses are needed prior to calibration, and the uncertainties analysis of results need to be evaluated after validation. Calibration and validation of complex models are a labor intensive and time-consuming process [96]. For application of complex models end users need expertise, sufficient training, and experience to apply these models correctly.

#### 2.3.1. AGNPS/AnnAGNPS

AGNPS (Agricultural Non-Point Source Pollution Model), initially developed by USDA-ARS cooperated with the Minnesota Pollution Control Agency and the National Resources Conservation Service (NRCS), is a single event-driven NPS pollution model [21,97]. The latest AGNPS version 5.0 was released in 2018 [98]. AnnAGNPS (Annualized Agricultural Non-Point Source Pollution Model) is the upgraded product of AGNPS and did not focus on a single rainfall event but evolved into a modeling system that can conduct watershed-scale, continuous pollutant load simulations at a daily time step. AnnAGNPS 5.0 appends a pesticide component to the model. The latest version of AnnAGNPS is v5.5 [99], which has a GIS-based DEM analysis program and a Windows-based input editor for writing and revising of input data.

AnnAGNPS can estimate the current or long-term effects of alternative management decisions on surface runoff, sediment, and nutrients loading on a daily time step from predominantly agricultural watersheds ranging from a few hectares to 300,000 hectares [21,100]. AnnAGNPS uses homogenous land areas as square cells or any hydrologically-based shape that represents similar soil types, land use and management, and climate to discretize a watershed. Water, sediment, nutrients, and pesticides are generated from those homogenous land areas and then routed through stream networks finally to the watershed outlet [101]. The model uses the SCS-CN equation to estimate surface runoff [102] and soil moisture content to calculate potential evapotranspiration, applies the RUSLE method to estimate sheet and rill erosion [103], and adopts the hydro-geomorphic USLE to predict sediment delivery of the stream [104]. Core components of the model include hydrology, soil erosion, sediment, and nutrient transport. The simulated nutrients include nitrogen, phosphorus, organic carbon, fertilizer, pesticides, and chemical oxygen demand (COD), and point source loads [97]. The primary inputs of the model include precipitation, soils, land use, agricultural management, upland and channel networks, point source pollution (e.g., animal feedlots, streambanks, construction sites). The model outputs runoff volume, peak flow rate, erosivity, upland and channel erosion, sediment delivery ratio, sediment enrichment ratio, mean sediment concentration, total sediment yield, and pollutant concentration on an event, monthly, or yearly basis.

AnnAGNPS has been widely used to estimate runoff water quality and NPS pollution around the world. Li, et al. [105] applied AnnAGNPS to simulate yearly streamflow and monthly nutrient loading in the Taihu Lake watershed, China. Their results showed that the AnnAGNPS model can acquire a satisfactory accuracy for annual streamflow simulation, and the accuracy of the nutrient loading simulation is moderate, and monthly nitrogen loading evaluation has higher accuracy than monthly phosphorus loading. Karki, et al. [106] to evaluate runoff, nutrients, and sediment for an agricultural watershed of 30.3 ha in East-Central Mississippi by applying the AnnAGNPS model. They indicated that AnnAGNPS can perform better for runoff evaluation than sediment and phosphorus load assessments on a longer time scale. The accuracy of the model prediction dramatically depends on high quality available data for calibration.

AnnAGNPS is flexible and reliable tool to evaluate the amount of runoff, sediment, and nutrient generated and transported throughout a watershed. It can help to identify critical source areas and delivery routes of NPS pollution, support the determination of BMP locations, and cost/benefit analyses. The predictions of sediment and nutrient loads perform better at larger monthly, seasonal or annual time scales, than shorter daily simulations [107]. However, AnnAGNPS assumes a constant nutrient loading rate for point source pollution simulation for the entire simulation period and does not account for spatial variability of rainfall [101]. AnnAGNPS does not track nutrients and pesticides attached to sediments in stream reaches from one event to the next event [100]. AnnAGNPS may underestimate daily streamflow during a dry period, as it does not account for the baseflow. Additionally, AnnAGNPS need intensive parameter inputs to support model simulations, which may lead to increased computing demand for parameter optimization, calibration, and validation.

#### 2.3.2. SWAT

SWAT (Soil and Water Assessment Tool), developed by Dr. Jeff Arnold for the USDA-ARS, is a continuous, semi-distributed, physically-based watershed model [20]. The SWAT model is regularly updated, SWAT2012 rev.670, at the time of this publication, was released in October 2018 [108].

SWAT can evaluate surface water, crop development, sediment, nutrient yield, pesticide transport, and the impact of climate change and land management based on hydrologic inputs in a complex, ungauged watershed under various soil, vegetation, and land use management conditions [109]. SWAT operates at daily or hourly time steps and can perform long-term, continuous-time simulation. Spatial scale of SWAT applications ranges from small size watershed to an entire continent [110,111]. Within this tool a watershed is divided into multiple sub-basins, then a sub-basin is further subdivided into hydrological response units (HRUs) where all land areas have homogeneous land use, soil characteristics, and slope combinations. In each HRU, hydrological components are calculated for surface water, soil, and groundwater [109]. SWAT uses an SCS-CN method or Green & Ampt infiltration method to determine runoff volume and applies a rational formula or TR-55 method to calculate the peak flow rate. Soil erosion generated by rainfall and runoff is computed with the Modified USLE equation [112]. Land use, soil, weather and topography are the required primary input to SWAT. The output of SWAT includes water volume that sums step and accumulated surface runoff and subsurface flow, sediment yield, soil water storage, evapotranspiration, and nutrients [113].

SWAT has been widely applied to many watersheds in the world, including hydrologic modeling, non-point source pollution control, surface or subsurface water quality evaluation, groundwater modeling, soil erosion prevention and control, the impact of land use management practices, and the prediction of hydropeaking [4,31,114]. According to the official SWAT literature database, the number of articles relevant to SWAT has exceeded 1300 from 2016–2019 [115].

SWAT can be applied to various spatiotemporal scales ranging from sub-daily to yearly, and from small watershed (e.g., 10 km^2^) to a river basin and even a continent and enables users to study the long-term environmental impacts. SWAT can obtain higher prediction accuracy while predicting on a yearly or monthly scale than on the daily scale [76]. Data needed to support SWAT simulations are readily available from various sources including governmental agencies. SWAT can also be applied to a watershed with scarce or no monitoring data [109]. SWAT has an active and influential online user community [116]. Alternative calibration and validation approaches have been developed to simplify the often time consuming and difficult calibration process [117] for example SWAT-CUP (SWAT-Calibration and Uncertainty Programs), an auto-calibration program for SWAT [95]. SWAT-CUP is available at [118]. SWAT assumes vegetation growth is insensitive to season change, which often causes a low accuracy of SWAT prediction in the dry season [119]. One solution is to divide wet and dry seasons during calibration and validation, which can efficiently improve SWAT simulation accuracy [120,121].

#### 2.3.3. SWMM

SWMM (Storm Water Management Model), developed by U.S. EPA, is a distributed, physically-based, dynamic stormwater runoff quantity and quality model [71]. The latest version of the model is 5.1, which is a Windows-based software released in 2018. User’s manual and software installable files of the model are available at [122].

SWMM is developed for the evaluation of a single rainfall event or long-term continuous rainfall-runoff processes from primarily urban areas. Users can use SWMM to: (1) design drainage system component; (2) calculate NPS pollutant loading for developing TMDL; (3) estimate BMP and low impact development stormwater controls to meet sustainable goals; (4) evaluate combined and sanitary sewer overflows; and (5) estimate the effect of land-use change on urban hydrology [123]. SWMM can track runoff quantity and quality for hourly or sub-hourly time steps. The spatial scale of SWMM applications varies from separate lots up to hundreds of acres. SWMM divides a watershed into a collection of homogeneous sub-catchments as a basic hydrological unit [71]. The model considers multiple physical processes such as surface runoff, infiltration, groundwater, flow routing, water quality routing, snowmelt, and surface ponding. SWMM applies hourly or more frequency rainfall data as input. SWMM requires inputs of buildup and wash off parameters to model stormwater quality, which produces pollutographs at any point in the watershed. Simulated pollutants include TN, TP, TSS, settleable solids, oil/grease, BOD, COD, total coliform, and other user-specified pollutants [124].

The SWMM model has been commonly employed in urban drainage flooding analysis, water quality and transport of contaminants [3], TMDL implementation plans [125], urbanization and climate change effects [7], and LID effectiveness [126]. Niazi, et al. [127] presented a synthetic overview of SWMM applications and gap analyses.

SWMM can efficiently simulate hydrological and contaminant transport in complex urban watersheds. SWMM can account for time-varying rainfall during the process of simulation [71]. The model has been continuously upgraded since 1971 through present [71]. One primary limitation of SWMM is that as an analytical tool (not a design tool), it does not simulate small outlets (e.g., manhole or inlets) loss directly, but rather can be aggregated [128].

#### 2.3.4. HSPF

HSPF (Hydrological Simulation Program-Fortran), jointly developed by the U.S. EPA and the USGS, is a comprehensive, continuous, physically-based watershed hydrology and water quality model [19]. The latest version 12.2 of HSPF can be downloaded via the BASINS model from [129]. This version is WinHSPF 3.0 that integrated into BASINS as a core module. The users can also access a standalone HSPF version 11.0 from USGS website [130].

HSPF is used to simulate water quantity and quality processes, conventional and toxic organic pollutants loads within a natural and developed watershed, and predicts nutrients fate and transport routing in-stream and well-mixed lakes and impoundments [19]. The simulated time scale of HSPF is from a few minutes to several hundred years by using time steps ranging from sub-hourly to daily. The spatial extent of HSPF application varies from a few acres to a large watershed (the Chesapeake Bay with roughly 160,000 km^2^) [131]. In HSPF, a basin is divided into land units that can reflect a homogeneous hydrologic and water quality response [132]. HSPF has three primary modules: PERLND, IMPLND, and RCHRES. The PERLND controls runoff and water quality from pervious areas; the IMPLND module simulates water quantity and quality on impervious land segments; the RCHRES module reflects the route of flow and water quality constituents from the PERLND and IMPLND modules [133]. HSPF needs continuous time-series records as input including precipitation, potential evapotranspiration, air temperature, wind, solar radiation, humidity, tillage practices, and point sources. Water quality processes simulation also needs chemical pollutant (e.g., pesticide or fertilizer) application data. HSPF outputs include flow rate, sediment yield, nutrients, pesticides, toxic chemicals, and other water quality variables [134].

An early HSPF application literature summary can be found at [135]. Kim, et al. [5] integrated HSPF with a maximum likelihood filter to improve water quality forecasts in the Kumho catchment (23,384 km^2^) in South Korea. Huo, et al. [136] used HSPF to evaluate nonpoint source water quality in the Feitsui reservoir watershed (303 km^2^) in Taiwan.

HSPF is a comprehensive watershed model for agricultural or urban areas. HSPF adopted the flexible module design, thus users can use different simulation algorithms (empirical or physical) to analyze NPS pollution processes based on how much data available [132]. HSPF is suitable for predicting yearly and monthly streamflow and sediment yield, except for the months with extreme weather conditions. Daily streamflow simulations are reasonable except for flood events. HSPF is able to adapt to a wide range of watershed conditions involving various surface water and groundwater quantity and quality problems at multiple spatiotemporal scales [137]. The limitation of HSPF is that model calibration process requires expertise and experience to determine appropriate parameters, currently available documentation provides no uniform parameter estimation guide [131]. The data requirement of the model is extensive [138]. Due to use of a nonlinear flow or storage-based equation in routings, HSPF cannot simulate an intense single-event storm or flood waves, especially for a large sub-basin and long channel [33].

#### 2.3.5. WAM

WAM (Watershed Assessment Model), developed by Soil and Water Engineering Technology (SWET), Inc., is a GIS-based, deterministic, physically-based watershed-scale hydrology and water quality model [43]. WAM works as an extension in the ArcGIS environment. The latest WAM supports ArcGIS 10.4.1. Users can download WAM and its associated documents from the link [139].

WAM can estimate the hydrology and water quality response of land-use changes and associated management practices within a complex watershed including agriculture/urban land uses, natural or man-made stream networks, looped flow networks, multiple hydraulic structures, springshed groundwater systems, and tidally influenced boundary conditions [43]. It can run on both daily and hourly time steps to estimate surface water flow, groundwater flow, and pollutant loads. In WAM, a watershed is discretized into many single cells that represent the basic unit of hydrology and water quality simulation. WAM integrates GLEAMS (Groundwater Loading Effects of Agricultural Management Systems) and EAAMOD (Everglades Agricultural Area Model) to simulate soil and plant processes that affect water quality [140]. The calculations of daily surface and subsurface flow, nitrogen and phosphorus concentrations take place in each cell. The model then routes the surface and subsurface flow from cells to estimate the flow and phosphorus levels throughout the watershed by using the Basin Land Area to Stream Routing model (BLASROUTE). Water quality variables include TN, TP, TSS, and BOD. Primary inputs of the model include land use, soils, topography, climate data, and point source data. The outputs include surface and groundwater flow, pollutant loads, a ranking of land use by load source, daily time series of flows and pollutants, and displays of different BMPs in table, graph, and map [43].

The applications of WAM include the simulation of constituents that are important to predict eutrophication processes in water bodies, analysis of the hydrological and water quality effects on rivers and lakes for management scenarios, view and estimation of the simulated flow and concentrations for every source cell and stream reach, and prioritization of BMPs zones [141]. Bottcher [142] built a Suwannee River watershed assessment (25,770 km^2^) model based on the early ArcView version WAM, displayed spatial loads of soluble N, soluble P, TSS, and BOD, and simulated flows, total P, and total N on the daily and seasonal time scale. Bottcher, et al. [140] applied WAM into a predominately agricultural watershed to hydrologic and hydraulic processes and NPS pollution loading simulation with 2025 and 2055 future scenario analysis. Corrales, et al. [13] used WAM in the northern Lake Okeechobee watershed (4150 km^2^) to evaluate total P load at the source area, streams, and outfall levels.

WAM can simulate complicated surface and subsurface hydrologic processes and nutrient loading. It can describe spatial and hydraulic details and is flexible to accommodate varied hydrologic, water quality, land and water management processes, and support scenarios analysis. WAM accounts for the pollution contribution of upland landscape with deep water tables, lands with shallow water tables with and without artificial drainage, and wetlands, urban areas, and mining sites [141]. However, the drawback of WAM is limited numbers of applications based in Florida and it requires intensive physical characterization data, which might be hard to obtain for some places. WAM cannot simulate small-scale and short-term storm event impact.

### 2.4. Modeling Systems

A modeling system uses the concept of multiple modules to independently maintain and apply separate model structures or information to carry out complex decision analyses or synthesis. It integrates databases, tools, techniques, and models into a GIS platform. These data, tools, methods, and models have a close linkage and work together to perform various environmental simulations and analyses at multiple time and space scales, and different modules comply with the same data exchange protocols. Thus, the modeling system comprehensively uses compatible data types, various tools, and different models to assist in systematically solving complicated watershed water quality and NPS pollution issues. It can serve as a multipurpose decision support platform. The modeling system can simulate watershed-scale, hydraulic, hydrologic, water quality, and NPS pollution issues. However, the data requirement and model computation is enormous. These models have a steep learning curve for users as many individual models and various watershed analysis tools are integrated. This can involve extensive pre-processing and postprocessing of data and output. The user commonly needs extensive training prior to developing or running these models.

#### 2.4.1. AGWA

AGWA (Automated Geospatial Watershed Assessment Tool), co-developed by the U.S. EPA, USDA-ARS, the University of Arizona, and the University of Wyoming, is a GIS-based distributed, light level hydrology modeling system [143]. AGWA underwent a number of upgrades from AGWA 1.5 for ArcView 3.x, AGWA 2.x for ArcGIS 9.x to AGWA 3.x for ArcGIS 10.x. The user’s manual, theoretical documentation, training, and software of AGWA are available at [144].

Using readily attainable GIS data, AGWA can evaluate the impacts of land-use change on soil erosion, water quantity and quality, and watershed-scale NPS pollution at different spatial and temporal scales, ranging from a small drainage area to a large size watershed [39]. AGWA facilitates the processes of parameterization, model execution and outcome visualization and packaged inside multiple sub-models such as RHEM (Rangeland Hydrology and Erosion Model), KINEROS2 (Kinematic Runoff and Erosion Model), KINEROS-OPUS, SWAT2000, and SWAT2005. RHEM is a rangeland soil erosion model by water for a single rainfall at hillslopes. RHEM is integrated into the overland flow component of KINEROS2. KINEROS2 is a physically-based, event-driven model that can simulate vegetation interception, soil infiltration, surface runoff, and soil erosion in small watersheds. KINEROS-OPUS is a combination of KINEROS and OPUS2 (not an acronym) with more sophisticated functionalities including simulations of runoff, sediment transport, nitrogen and phosphorus cycling, and chemical transport processes. Comparatively, SWAT is a hydrology and water quality model for long-term watershed modeling, and details on SWAT have been presented in Section 2.3. The modeling inputs of AGWA include DEM, land use, soil, and precipitation data. The outputs of the KINEROS sub-model include channel infiltration, plane infiltration, runoff volume, sediment yield, peak flow, channel erosion, and sediment discharge. The outputs of SWAT sub-model have runoff volumes, evaporation, percolation, transmission losses, water yield, sediment yield, and nitrate and phosphorous in surface runoff [145].

The applications of AGWA involve watershed management, water resource assessment, land-use change detection, and ecology evaluation. Yang and Li [146] employed AGWA-SWAT and KINEROS to evaluate the hydrological response of urban development in a Panther Creek watershed (94.2 km^2^) with future land-use scenario analysis. Jones, et al. [15] used AGWA-KINEROS2 to estimate sediment delivery on the post-fire land cover to Strontia Springs Reservoir during a rainstorm with alternative scenarios.

AGWA provides a repeatable method to facilitate the setup and execution of multiple sub-models. AGWA supports future scenario analyses, decision assist, and hydrology and soil erosion simulations at different spatiotemporal scales [143]. Further, AGWA can also supply a quick post-fire watershed evaluation by replacing the part of an existing land use map with a burn severity map [147]. However, AGWA does not integrate within the latest version (SWAT2012 or SWAT+) of SWAT. The tool does not consider the data acquirement component, so users cannot collect online data via AGWA.

#### 2.4.2. BASINS

BASINS (Better Assessment Science Integrating Point and Nonpoint Sources), developed by the U.S. EPA, serves as a multipurpose environmental analysis system [148]. The latest version 4.1 of BASINS was released in 2013. It is based on a user-friendly open-source MapWindow GIS and is available at [129].

BASINS is suitable for watershed management, water quality analysis and TMDLs development, and integrates environmental data, analysis tools, and various watershed and water quality models. BASINS facilitates examination of environmental issues, support analysis of watershed systems, and provides a platform for inspecting management alternatives [134,149]. As a comprehensive watershed model framework, BASINS integrates several watershed models such as HSPF, SWAT, SWMM, GWLF-E, PLOAD (Pollutant Loading Estimator), and instream and water quality models such as AQUATOX and WASP (Water Quality Analysis Simulation Program) as plug-ins and some analysis tools such as manual/automatic watershed delineation, land use reclassification, lookup tables, shapefile editor, PEST (the Parameter Optimization Program), time series functions, CAT (Climate Assessment Tool), GenScn (GENeration and analysis of model simulation SCeNarios), and WDMUtil (Watershed Data Management Utility) into a unified GIS interface [134,148]. The overarching objectives of BASINS are to: (1) identify and prioritize water bodies; (2) evaluate magnitude and potential significance of point and nonpoint source pollution; (3) simulate point source and nonpoint source nutrient loadings and fate and transport processes; (4) evaluate the relative value of potential control strategy; and (5) visualize environmental conditions to the public through tables, graphs, and maps [148]. Core data in BASINS includes four main types: base cartographic data, environmental background data, monitoring data, and point source data. The input data include DEM, land use, soil, weather data, and point source data, and output files include maps, graphs, and tables summarizing point and non-point source pollution in a watershed [148].

BASINS has been used to develop solutions for solving real-world problems in the past two decades. Saleh and Du [134] compared the application results of BASINS-SWAT and HSPF in the Upper North Bosque River watershed. HSPF performed better than SWAT in terms of evaluating daily flow and sediment; however, SWAT was a much better predictor for simulating daily and monthly nutrient loadings. Crossette, et al. [149] applied BASINS-HSPF to the data-scarce Shebelle watershed (2400 km^2^) in central Ethiopia and presented the detailed steps of the BASINS application.

BASINS is a multipurpose environmental model system used to conduct watershed hydrology and water quality studies and develop TMDLs for water quality impaired water bodies [150]. The model system facilitates watershed and water quality studies through decreasing data collecting and processing time, reducing execution steps, and minimizing error caused by incompatible data format [149]. Due to integrating different models, BASINS can simulate water quality and NPS pollution issues at various spatiotemporal scales. Moreover, the model can analyze and develop TMDL guidelines that meet the need of the Clean Water Act. However, BASINS has a steep learning curve because of the involvement of environmental theory and technical knowledge.

#### 2.4.3. WMS

WMS (Watershed Modeling System), developed by AQUAVEO Inc., is a comprehensive GIS-based modeling system for watershed hydrology and hydraulics [151]. The latest version of WMS is 11.0 and is available at [44]. WMS is not a public domain software.

WMS is designed to simulate hydrologic, hydraulic, storm drain, sanitary sewer, water distribution, and NPS pollution processes, including almost all phases of hydrology and hydraulics. WMS integrates eight modules under one GIS interface, including terrain, drainage, map, hydrologic, hydraulic (river), GIS, 2D grid, and 2D scatter point. Each module corresponds to one of the primary data types or modeling environments supported by WMS. WMS supplies a GIS-based framework to operate different models such as HEC-1, HEC-HMS, NSS, TR-20, TR-55, Rational Method, OC (Orange County, California) Rational, OC hydrographic, HSPF, SWMM, XP-SWMM, SMPDBK (Simplified Dam-Break Model), GSSHA (Gridded Surface Subsurface Hydraulic Analysis), CE-QUAL-W2, HY-8, HY-12, Hydraulic Toolbox, and EPANET [17]. The model integrates national streamflow statistics, supports conversion and comparison of the results from different sub-models, displays a comparison between observed and simulated hydrographs, provides terrain surface viewing, and exports images for reports and presentations [44].

WMS has been applied to watershed management [152], flood hazard analysis [153], water quality evaluation, and groundwater simulation [29]. Erturk, et al. [152] used WMS 7.1 for watershed management and NPS pollution modeling in Koycegiz Lake-Dalyan Lagoon watershed (1200 km^2^) located at the southwest of Turkey. WMS supports a flexible watershed delineation method. For example, users can also control watershed boundaries created by WMS. The model system has detailed documentation along with an active user community [44]. However, it is not a public domain software, and users need to acquire a WMS license for use.

## 3. Current Challenges within NPS Pollution Models

### 3.1. Model Selection

As described above, the NPS pollution model selection is rather challenging for model users based on the fact that numerous different watershed models are available currently. The users have to consider multiple factors while selecting an appropriate model for a specific project, including the nature of watershed issues that need to be solved, the processes that are interested to simulate, the complexity of the model, desired spatial and temporal scales, data requirement, expected output and simulation accuracy, user’s knowledge and skills, and the budget of the project.

The desired output or management information needed should dictate the selection of a particular model. Some models are appropriate for estimating numerical values with minimal input while others are designed to inform complex decisions with intensive data input from multiple sources. For example, some tools may provide a quantitative estimate of the areal average annual pollution load from a watershed with existing data, while others are designed to inform a management practice or scenarios such as optimization of BMPs or determination of TMDLs for a watershed. A simple model or a medium complexity model may provide sufficient results for a specific unknown value in the former situation, but the latter problem might need to adopt a complex model or a modeling system combined with expert practitioners to apply multiple data inputs and management insight to generate meaningful or useful model output. The classification of NPS pollution models described here is a straightforward way to begin the model selection process and should help users choose the appropriate model or tool based on an understanding of how the models function and what the models require. Moreover, a detailed categorization and identification of individual model structure and function is helpful for those tasked in model selection in practice, but also provide insight to the manager charged with providing useful decision-making information. Table 2 lists the main features of each model discussed in the article to identify model selection criteria and compare different models.

Each NPS pollution model has unique function and characteristics for specific purposes. The more particular processes of simulation users expect, the less the range of model selection is. If a user needs to know the environmental contamination of mercury in a watershed, he/she might not face many difficulties from model selection since few models integrate mercury transport/transformations algorithm and codes. WARMF is a good option for that specific simulated objective. However, users need to be aware that each model is a simplification of the real world. Any simplification implies some physical processes are omitted in the model design. If the ignored processes are of significant importance for modelers, the user should investigate alternative approaches or at least be aware of the limitations of the selected model or tool.

Generally, the NPS model performs better on smaller spatial scales, and longer time steps (i.e., monthly or yearly) [154]. Some NPS pollution models can conduct an event-driven pollution simulation such as AGNPS, SWMM, and SLAMM; while others are more appropriate for the long-term continuous simulation including AnnAGNPS, GWLF, LSPC, L-THIA, N-SPECT/OpenNSPECT, WARMF, and SWAT. Few models can conduct both short- and long-term NPS pollution simulation such as HSPF, BASINS, WMS, and WAM. A lumped parameter, empirical model can quantitively calculate long-term average gross NPS pollution loads, but it does not consider the fate and transport, and spatial route of nutrients loads. A distributed, physically-based model presents the spatial distribution of pollutants, and can account for fate and transport of nutrient, but needs a lot of input parameters. Therefore, users should select a suitable watershed model in light of the watershed size and time duration of a simulated event.

### 3.2. Spatial and Temporal Considerations

Spatial and temporal scales affect many aspects of NPS pollution model applications such as model selection, watershed discretization, data pre-processing, as well as simulation accuracy [155]. Firstly, model selection will be determined mostly by the space and time scale of the model application. From the spatial perspective, the main transport route of runoff, sediment and nutrients is dominant by river networks system at a river scale basin. A large size watershed is less sensitive to short-duration, high-intensity rainfall, and the model structure will determine how well the model simulates these patterns with given lags and routing. In contrast, a small watershed has a sensitive response to high intensity, short duration rainfalls because overland runoff and streams control transport route of sediment and nutrients transportation and may be less sensitive to model structure or parameterization [147]. From the temporal point of view, NPS pollution processes may occur at any time scale (i.e., event, daily, monthly, or yearly), therefore different watershed-scale NPS models meet the requirement of the simulation of NPS pollution scenarios at various time scales. An event-driven NPS pollution model is typically used for the simulation of a short-duration, intensive rainfall process. A physically-based, long-term continuous-simulation NPS pollution model can estimate various hydrologic processes under multiple time scales, typically execute on a time span range from a few minutes to hundreds of years and its outcome can present a time-series of runoff, sediment, and pollutants loadings. Spatial and temporal considerations of NPS pollution model are discussed in detail by Baffaut, et al. [156].

Secondly, watershed space can be discretized into a grid cell, any shape subarea, HRU, or sub-watershed that represents the smallest spatially computed unit of the model. AnnAGNPS users are recommended to use a cell size of 40 acres to operate the model when the size of a watershed exceeds 2000 acres [157]. Much of the measured data related to NPS pollution are obtained from observations and artificial experiments at a laboratory or field, which is useful to verify understanding of NPS pollution underlying processes. However, when these experimental data are applied to watershed-scale NPS pollution simulation, it results in additional model uncertainty because they cannot correctly reflect the pattern of NPS pollution at a larger scale. If the selected scales do not match between observed data and model output variables, then upscaling or downscaling method might be applied to solve this problem. Upscaling refers to aggregate data from a smaller scale to represent a larger scale; and downscaling is used to disaggregate data at a larger scale to suit a smaller scale [158].

### 3.3. Calibration and Validation

The primary purpose of calibration is to build a mapping relationship between an NPS pollution model and the physical real-world. The target of calibration is to minimize the model error between simulated results and observed data by adjusting selected input parameter values. The calibration process will eventually find the optimized parameter combinations that make the model obtain higher accuracy and less uncertainty. Validation has the same process with calibration except for using an independent dataset from a different period and keeping calibrated-well parameters unchanged [159]. The purpose of validation is to assure that the calibrated model can produce properly evaluated results under similar hydrological conditions with calibration.

Simple models do not require calibration and validation processes (i.e., L-THIA, N-SPECT) since these models have been sufficiently verified at a specific region during the processes of model development. Simple models are typically used as a quick evaluation tool to present a relative rough estimation. Validation of these models is recommended when they are applied to other areas where they have not been verified. Lim, et al. [160] developed an automatic calibration system to search the optimized CN combinations while estimating runoff and pollutant in the Litter Eagle Creek watershed in Indiana. The authors found that the simulated results of hydrology and water quality can be significantly improved after calibration. Simple models are limited to calibrate SCS-CN and/or EMC parameters due to the simplicity of the model structure. On the other hand, the execution of complex NPS models or modeling system (i.e., AnnAGNPS, SWAT, SWMM, SWAT, WAM, BASINS, WMS) involves many parameter inputs, but most of the parameter values are not exactly known due to spatial variability and direct measurement is unavailable [161]. Thus, the model needs to obtain these parameter values through calibration before it may be practically applicable as a decision-making tool.

The modeler conducts parameter calibration processes by adjusting parameter value and running the model repeatedly. The calibration methods of a model have typically two: manual or automatic. The manual calibration depends on the experience of the modeler because it is difficult to search and try all parameter combinations. In most cases, the manual calibration process will cease once statistics from the objective function satisfied the target set by the modeler before running the model. The operation of manual calibration is complicated for non-expert users. In contrast, automatic calibration is an iterative procedure that can try more parameter combinations by using a grid search or other algorithms and setting searching value range of different parameters. Some standalone and professional automatic calibration procedures are developed for the specific model to facilitate the application of NPS pollution models and shorten the time of calibration. SWAT Calibration and Uncertainty Programs (SWAT-CUP) is a standalone, robust, and public software program to automatically calibrate parameters from SWAT model, which integrates Sequential Uncertainty Fitting 2 (SUFI2), Generalized Likelihood Uncertainty Estimation (GLUE), Markov Chain Monto Carlo (MCMC), Particle Swarm Optimization (PSO) and Parameter Solution (ParaSol) procedures [162]. The number of parameters that can be calibrated by SWAT-CUP is over 700. A detailed introduction of various calibration procedures with SWAT-CUP was provided by Yang, et al. [163]. SWAT-CUP facilitates sensitivity analysis, calibration, validation, and uncertainty analysis that almost involves the entire post-processing of SWAT simulation. A more detailed discussion on the use, calibration and validation of the SWAT model is provided by Arnold, et al. [109]. Additionally, HSPF Parameter (HSPFParm) database support software was designed for the HSPF model to identify initial value and expected ranges of parameters before running HSPF calibration progress. Expert System for Calibration of HSPF (HSPEXP) was developed to estimate statistical error for HSPF simulation of each time input parameter, which also provides specialist advice about optimized parameter combinations to improve the calibration [132]. According to Duda, et al. [131], HSPEXP procedure only works for manual hydrology calibration, not for water quality calibration, and does not consider snow simulation.

Notwithstanding, most NPS pollution models need to be calibrated but there are few professional and standalone procedure like SWAT-CUP and HSPEXP to assist the calibration process of other models. Moreover, even with the presence of an automatic calibration method, the model calibration is still a time-consuming process because of multiple involved procedures such as the preparation (formatting) of calibration file, parameter sensitivity analysis and iterative algorithm running. It should be noted that the calibration is often a hierarchical process beginning with flow volume, followed by sediment loads, and finally followed by NPS pollutant loading rates [131]. In practice, the calibration of nutrients concentration or loads is typically challenging to be accurate because of the existence of model uncertainty as well as a lack of observed pollution data in practice. According to Moriasi, et al. [158], model performance can be evaluated as ‘satisfactory’ if PBIAS is in range of ±70% for N and P while other statistical indicators can also satisfy the criterion. Detailed model calibration and validation strategies and steps are beyond the scope of this review article, but more calibration and validation information can be obtained from published literature [90,109,131,161,164].

### 3.4. Uncertainty Analysis

Uncertainty is a natural and inherent deficiency of models. Uncertainty of NPS pollution models comes from three aspects: structural uncertainty, data uncertainty, and parameter uncertainty [164]. The conceptual design of the model is simplified so it brings structural uncertainty. These simplifications or assumptions neglect some physical processes occurring in a watershed. For example, wind erosion and landslides processes are typically ignored at NPS pollution models. Additionally, it causes uncertainty of simulated results because the mechanism and process of pollution fate and transport in the stream might not have been entirely and correctly described in currently available NPS pollution models. Structural uncertainty also involves some unknown occurrences to a modeler although processes affecting water quality have been included in the model such as upstream reservoirs, dam operation, and farm fertilization [162]. In addition to structural uncertainty, some uncertainties come from errors of input data such as DEM, soils, rainfall, temperature, land use and point measurements, these data are used in complex models, but their values are rarely exactly known. For example, spatial uncertainty of rainfall could be large different especially in mountain areas, as well as the resolution of DEM and measuring error of point data have a significant impact on model uncertainty [165]. Finally, parameter uncertainty reflects the parameter non-uniqueness issue. Clearly, the optimized solution for model calibration is never unique and different parameter combination may output very similar simulation results [95]. Therefore, it is crucial for the modelers to clearly understand the intrinsically physical meanings and value ranges of major parameters at specific research areas. Furthermore, uncertainties from the interaction between model structure, input data, and parameters cause the more complex uncertainty of evaluated results from NPS pollution simulation.

Uncertainty analysis is a process to estimate the effect of structural uncertainty, data input and parameters on model results [164]. The evaluation of model structural uncertainty can be obtained by analyzing statistic characteristics from the error series of the objective function under the assumption without input and parameter uncertainty existing. The typical method is to compare the simulated error series of the same study area with different models [137]. Input uncertainty involves the spatial distribution of rainfall, the resolution of DEM and measurement errors of point data. The impact of rainfall input uncertainty on model output focuses on the adjustment of the density of rainfall stations and their spatial distribution by comparing different spatial interpolate methods. The influence of DEM resolution uncertainty on model output is studied by changing DEM grid size and observing the change of extracting terrain parameter and model output. Not much attention has been paid on how point data measurement error affects the model uncertainty so far [166]. The research method is to treat point data input as a random input, to get a prior probability distribution of measured values, which is then used to obtain the posterior probability distribution through statistic likelihood function by comparing this distribution to get model uncertainty due to input data errors. Another method is to add interference random number that follows with normal distribution into the original input series to observe the difference of model output. Currently, more researches focus on the impact of parameter uncertainty on model results. The research methods of parameter uncertainty involve parameter sensitivity analysis and probability analysis [163]. Two conventional methods of sensitivity analysis are: local (or One-at-a-time, OAT) and global (or All-at-a-time, AAT). OAT creates variability in one input parameter to determine the output result, while fixing remaining other parameters. In contrast, AAT can show the variance of all input parameters, with output results uncertainty averaged over the input parameters. The probability analysis method is used to describe the parameter uncertainty of a model. Since uncertainty is a structural and inevitable characteristic of NPS pollution models, uncertainty analysis is an important step that helps appropriately select and apply a model. It should be mentioned that in model evaluations, there exists an uncertainty range that shows to which extent the model can capture the observed signal.

## 4. Summary and Future Research Direction

This review article summarizes 14 watershed-scale NPS pollution models associated with essential model characteristics such as intended use, components/capabilities, model structure, applicable land use, spatial and temporal scale, availability as well as the strengths and limitations for each model. The categorizations, simple model (L-THIA, N-SPECT/OpenNSPECT), medium complexity model (GWLF, LSPC, SLAMM, and WARMF), complex model (AGNPS/AnnAGNPS, SWAT, SWMM, HSPF, and WAM), and modeling system (AGWA, BASINS, and WMS), are used to guide users to identify the most appropriate model for his/her project. A simple model relies on minimal data, does not need rigorous calibration, and typically serves as a quick screening tool for NPS pollution evaluation. A medium complexity model is generally applicable topoint and nonpoint source pollution problems in diverse watersheds. These medium complexity models require detailed data input but minimal calibration requirements. They are commonly used as a compromise between simple models and complex models. A complex model can represent the complex processes of NPS pollution at different spatial and temporal scales and requires intensive data input. Parameters sensitivity analysis, calibration, validation, and uncertainty analysis need to be conducted while using complex models. A modeling system works as a multipurpose decision support platform that can provide an entire solution of watershed NPS pollution issue, but users need to possess profound professional knowledge and technique for appropriate use.

Omission or absence of any of the many useful and valuable models from our review should not be interpreted as a judgement and we encourage the readers to investigate beyond the limited number of models discussed here, including a recently published government report that describes popular models based on their publication frequency in scholarly literature (see [167]).

Model selection is a challenging task and is limited to different factors such as the nature of the watershed issue, the processes that need to be simulated, desirable spatial and temporal scale, data requirement, project cost, and so on. Table 2 is helpful to model selection by demonstrating a detailed classification and comparison of multiple models with key characteristics. Among these models, AGNPS, SWMM, and SLAMM are applicable to an event-driven simulation; AnnAGNPS, GWLF, LSPC, L-THIA, N-SPECT/OpenNSPECT, WARMF, and SWAT suited for a long-term continuous simulation; HSPF, WAM, BASINS, and WMS can conduct both short- and long-term simulation.

It is essential for most models to be well-calibrated and validated before they can be practically applied for the planning and management of watershed water resources. The manual calibration method is not recommended to non-expert users. Automatic calibration can significantly reduce model uncertainty, shrink model calibration time, and quickly find optimization parameter combinations. However, most NPS pollution models do not have a standalone and professional automatic calibration procedures for model calibration, validation, and uncertainty analysis. Therefore, it will be a promising direction to develop a universal, cross-model, and automatic calibration procedure that integrates advanced algorithms of parameter search.

Since uncertainty is an inherent and inevitable characteristic of all models, uncertainty analysis is still a frontier area in NPS pollution model future research. Current studies mainly focus on parameter uncertainty, so it should be strengthed on studies of reducing model uncertainty that stem from the model structure and data input, including to develop new research measures and innovate uncertainty evaluation techniques. Additionally, the future direction of NPS pollution models will involve strengthening the mechanism study of NPS pollution processes, especially for some specific pollutants, making the mathematic model more correctly describe the pollution processes. Furture work will also invovle developing new methods to improve the application accuracy of NPS pollution models, particularly in areas with limited data.

## Figures and Tables

**Table 1.** Characteristics of different NPS pollution model types.

Model Type	Strength	Weakness
**Simple Models**	• Minimal data requirement; • Estimated methods simply based on tire empirical or statistical relationships; • No mandatory calibration needs; • Suitable for long-term average pollutant load evaluations in a moderate to large watershed; • A quick streening tool	• Do not consider the spatial route of NPS pollution; • Cannot simulate pollutants fate and transport; • Coarse estimation accuracy of nutrient loads; • The number of simulated pollutants is limited

**Medium Complexity Models**	• Detailed data input needs; • Considers fundamental physical processes of surface or subsurface water and combine the empirical relationships for nutrient loads; • Can assist in the water quality criteria (e.g. TMDL) development • Minimal calibration effort and relatively easy to use	• Restricted simulation capability for water and sediment movement; • Ignores or over-simplifies pollutants fate and transport in streams; • Limited inclusion of spatial and temporal dynamic processes of pollutants generation and transport

**Complex Models**	• Provides rigorous description of flow, sediment, and nutrient loads and processes; • More pollutants may be simulated beyond nutrients or sediment; • Can express the pollutant fate and transport processes; • Flexible and wide range of simulation at diverse spatial and temporal scales	• Intensive data input requirements; • Laborious parameterization, calibration, and validation processes; • Requires analysis of uncertainty and careful interpretation of results; • Steep learning curve or training needed for model application

**Modeling Systems**	• Integrates databases, models, and tools to facilitate applications in large-watersheds; • Supply a more holistic solution for watershed hydraulics, hydrology, water quality, and pollution issues; • May serve as a multipurpose decision support platform	• Large data requirement, complex system structure, and computationally demanding; • Steep learning curve; • User need to possess profound professional knowledge and skill to drive and interpret the system running and interpret the outcome

**Table 2.** Main features of water quality and NPS pollution models.

	Model Name	Intended Use	Land Use/Input Parameters	Spatial and Temporal Scale	Simulated Pollutants	Strengths	Limitations	Software Information (Developer/Provider, Latest Version, Cost, and Link)
**Simple Model**	L-THIA	Estimate the long-term effects of land-use changes on runoff, groundwater recharge and NPS pollution	From non-urban areas to urban areas Lumped	A large size watershed, state or province Primarily long-term or single event	Sediment, TN, TP, nitrate, dissolved TP, metals, BOD	A quick screening tool for NPS pollution and water quality assessment [22]; Easy and free-to-use for the public [59]; A modest effort to prepare input data [56]	Average annual runoff volume and pollutant loading output [10]; Not account for the spatial variability of runoff, sediment and pollutant transport [36]	Purdue University; ArcL-THIA for ArcGIS 10.1; Public; https://engineering.purdue.edu/~{}lthia/ or http://npslab.kongju.ac.kr/#service
	N-SPECT or OpenNSPECT	Examine relationships between land cover, nonpoint source pollution, and erosion; Evaluate nearshore ecosystem health	Agricultural and urban areas Lumped	Medium-to-large nearshore watersheds Primarily Long-term or event-driven rainfall	Sediment, TN, TP, lead, and zinc	A flexible evaluation tool for NPS pollution, sediment, and soil erosion [41]; OpenNSPECT is a free, easy-to-use; Requires minimal data input [41]	Model structure is simple; Does not include spatial routing and processes of runoff, sediment, and pollution loads [67]	NOAA; N-SPECT/ OpenNSPECT 9.X; Public; https://coast.noaa.gov/digitalcoast/tools/opennspect.html
**Medium complexity Model**	GWLF	Estimate runoff, sediment and nutrient loadings; assists TMDLs development	Agricultural and urban areas Distributed/lumpe	Medium complexity or complex watershed d Long-term simulation with a daily time step	Sediment, dissolved and solid-phase TN, TP	Can be applied to an ungauged watershed; Modest data requirements [23]; Less complexity compared with SWAT, HSPF [69]; A compromise between an empirical model and complex physically-based models [69]	Not suitable for large watersheds or spatial variation dependent on channel routing [76]; Accuracy of GWLF is more dependent on the calibration processes than SWAT [11]	Pennsylvania State University; AVGWLF; Public; https://wikiwatershed.org/help/model-help/mapshed/
	LSPC	Evaluate hydrology, erosion, sediment transport, and water quality processes	Agricultural and urban areas Lumped	From small to large size, complex watershed Long-term simulation with a daily time step	Sediment, TN, TP, DO, BOD	Consider both upland contributing areas and receiving streams [40]; Dynamically modeling flow, sediments, nutrients, metals, and other pollutants from pervious and impervious lands and waterbodies; Developed for applications of mining and TMDLs formulation [77]	Does not allow for multiple sub-basins to connect to a single reach; Cannot manage complex groundwater routing, nor simulate surface-groundwater interactions [79]	Tetra Tech Inc.; LSPC 5.0; Public; https://github.com/USEPA/LSPC-Loading-Simulation-Program
	SLAMM	Identify urban pollutant source areas and assist urban stormwater management planning	Urban Lumped	Medium size urban watershed Event-based continuous simulation	Particulate solids, TN, TP, TKN, COD, chromium, copper, lead, zinc, fecal coliform bacteria	Built on actual field observations, which make the model is better to apply in practice [88]; Focus on small storm hydrology and particulate washoff [24]	Does not consider the processes of snowmelt and baseflow [87]; Does not consider instream processes that can remove or transform pollutants [85]	Robert Pitt, John Voorhees; ArcSLAMM for ArcGIS 10.1; Proprietary; https://www.usgs.gov/software/winslamm or http://winslamm.com/
	WARMF	A decision support system for watershed analysis and TMDL calculation, allocation, and implementation	Agricultural and urban areas Distributed	Any river basin Short/long-term	Sediment, pesticides, TN, TP, DO, BOD, pathogens, metals (Al, Fe, Zn, Mn, Cu, Hg from acid mine drainage), coliform bacteria, 3 algal species, periphyton	Calculate TMDL using a bottom-up approach [90]; Links catchments, river segments, and lakes to form a seamless model; Can be applied to acid mine drainage, mercury pollution, and on-site wastewater system [89]; Accounts for the source controls of atmospheric deposition, nonpoint and point source loads [42]	Does not consider a tile drainage system; Cannot model deep groundwater aquifer or quality; The subsurface flow component is simple [93]	System Water Resources, Inc; WARMF 5.0; Proprietary; http://systechwater.com/warmf_software/software-access/
*Complex Model*	AnnAGNPS	Evaluate NPS pollution and compare the effects of implementing various alternative conservation over time	Agricultural Distributed	A large watershed Long-term, continuous simulation with a daily time step	Sediment, TN, TP, pesticide, organic carbon, fertilizer, COD, point source loads	Simulate long-term sediment and chemical transport from ungagged agricultural watershed [97]; No preset limit on the number of cells, reaches, or length of simulation period [98];Flexible data input	Does not track nutrient and pesticides attached to sediment in-stream from one event to the next event [98]; Data-intensive; Point source loads are limited to constant loading rates for the entire simulation period [100]	USDA-ARS; AnnAGNPS 5.5; Public; http://go.usa.gov/KFO
	SWAT	Predict the effects of alternative land use management practice on water, sediment, crop growth, nutrient cycling, and pesticide	AgriculturalQuasi-distributed	From a small watershed to a continent Long-term continuous simulation with from sub-daily to yearly time step	Sediment, TN, TP, pesticides, bacteria, organic carbon, DO, BOD	Applied widely for various spatial and temporal scale watershed in the world [20]; Data is readily available from government agencies [109]; Used to a watershed with scarce or no monitoring data [113]	May not be appropriate to predict extreme hydrologic events [113]; Is not designed to simulate detailed, single-event flood routing [117]; Does not consider the impact of a season change on vegetable growth [76]	USDA-ARS; SWAT2012; Public; https://swat.tamu.edu/
	SWMM	A physically-based, dynamic, continuous urban stormwater runoff quantity and quality model	Urban Distributed	From single lots to hundreds of acres complex watersheds; Long-term continuous simulation with hourly or more frequent weather input or for a single event	Suspended solids, TN, TP, washoff loads, zinc, buildup, washoff	A prevalent model for primarily use in urban areas; Efficiently simulate hydrology and contaminant transport [71]; Model complex storm drain system with backwater effects [123]	Is not a storm design tool; Cannot model manhole or inlet loss directly [128]	EPA; SWMM5.1; Public; https://www.epa.gov/water-research/storm-water-management-model-swmm
	HSPF	Comprehensive watershed hydrology and water quality model for conventional and toxic organic pollutants	Agricultural and urban areas Distributed	From a few acres to a large watershed A few minutes to several hundred years with sub-hourly to daily weather input	Sediment, pesticides, TN, TP, BOD, phytoplankton, zooplankton, DO, pesticides, fecal coliforms, conservatives, ammonia, nitrate-nitrite	Prevalent, sophisticated, and applied widely in the world; A flexible solution of various surface and subsurface water quantity and quality problem at multiple spatiotemporal scales [19]	Data-intensive; Require a lot of parameters input [131]; Time-consuming calibrate and validate; May not be appropriate for extreme flow events [33]	EPA, USGS; WinHSPF 3.0; Public; https://www.epa.gov/ceam/basins-download-and-installation
	WAM	Evaluate environmental effects of various land-use changes and management practices on surface and subsurface hydrology and pollutant loads	Agricultural and urban areas Distributed	From a small to extremely complex large watershed Long-term continuous simulation with a daily or hourly time step	TSS, BOD, TN, TP, pesticide	Represent spatial and hydraulic details Flexible accommodate varied hydrologic, water quality, land and water management processes [43]; Consider upland landscape with deep water tables, land with shallow water table with and without artificial drainage [141]	Not good at simulating small-scale and short-term storm event impact [43]; Simplified instream water quality processes [43]; Data-intensive	Soil and Water Engineering Technology, Inc.; WAM Toolbar for ArcMap 10.4.1; Proprietary; http://www.swet.com/wam-for-arcmap-100
**Modeling System**	AGWA	A multipurpose hydrologic analysis system that integrated several sub-models	Rural Distributed/lumped	From small watershed- to basin- scale From single storm event to long-term continuous simulation	Sediment, TN, TP	A light-level modeling system Provides a repeatable method to facilitate the setup and execution of multiple sub-models [143] Predict runoff and erosion rates on rangelands [39]; Conduct rapid, post-fire watershed assessment [147]	Does not integrate the latest SWAT version; Do not include data online acquirement component	USDA-ARS, EPA, the University of Arizona, the University of Wyoming; AGWA 3.X; Public; https://www.tucson.ars.ag.gov/agwa/downloads/
	BASINS	Multipurpose environmental analysis system for watershed management, water quality analysis and TMDL development	Agricultural and urban areas Mixed	Varying	Sediment, pesticides, TN, TP, BOD, phytoplankton, zooplankton, DO	Facilitated watershed and water quality studies through decreasing data collecting and processing time, reducing execution steps, and minimize error caused by incompatible data format [148]; Simulate water quality and NPS pollution issues at various spatiotemporal scales [149]; Analyze and develop a TMDL standard and guidelines [134]	A steep learning curve because of involving much environmental theory and technical knowledge	EPA; BASINS 4.1; Public; https://www.epa.gov/ceam/basins-download-and-installation
	WMS	Simulate hydrologic, hydraulic, storm drain, sanitary sewer, water distribution, and NPS pollution processes	Agricultural and urban areas Mixed	Varying	Sediment, TN, TP, organic carbon, DO, BOD, algae, ammonium	Facilitate various sub-models’ execution; Flexible watershed delineation method [151]; WMS match the terrain data with the watershed delineation according to user’s expert knowledge [44]	Is not a public domain software; The number of applications is inadequate until the present	AQUAVEO Inc; WMS 11.0; Proprietary/Free trail; http://www.aquaveo.com/downloads?tab=3#TabbedPanels

Note: TN: total nitrogen; TP: total phosphorus; TSS: total suspended solids; DO: dissolved oxygen; COD: chemical oxygen demand; BOD: biochemical oxygen demand; TKN: total Kjeldahl nitrogen.

## References

[1] Niraula R; Kalin L; Srivastava P; Anderson CJ Identifying critical source areas of nonpoint source pollution with SWAT and GWLF. Ecol. Model. 2013,268,123–133.

[2] Liu R; Xu F; Zhang P; Yu W; Men C. Identifying non-point source critical source areas based on multi-factors at a basin scale with SWAT. J. Hydrol. 2016, 533, 379–388.

[3] Tuomela C; Sillanpaa N; Koivusalo H. Assessment of stormwater pollutant loads and source area contributions with storm water management model (SWMM). J. Environ. Manag. 2019, 233, 719–727. 10.1016/j.jenvman.2018.12.061 30641420

[4] Mittelstet AR; Storm DE; White MJ Using SWAT to enhance watershed-based plans to meet numeric water quality standards. Sustain. Water Qual. Ecol. 2016, 7,5–21.

[5] Kim S; Seo D-J; Riazi H; Shin C. Improving water quality forecasting via data assimilation—Application of maximum likelihood ensemble filter to HSPF. J. Hydrol. 2014, 519, 2797–2809.

[6] Fan M; Shibata H. Simulation of watershed hydrology and stream water quality under land use and climate change scenarios in Teshio River watershed, northern Japan. Ecol. Indic. 2015, 50, 79–89.

[7] Huong HTL; Pathirana A. Urbanization and climate change impacts on future urban flooding in Can Tho city, Vietnam. Hydrol. Earth Syst. Sci. 2013,17,379–394.

[8] Gitau M; Gburek W; Bishop P. Use of the SWAT model to quantify water quality effects of agricultural BMPs at the farm-scale level. Trans. ASABE 2008, 51,1925–1936.

[9] Strauch M; Lima JE; Volk M; Lorz C; Makeschin F. The impact of Best Management Practices on simulated streamflow and sediment load in a Central Brazilian catchment. J. Environ. Manag. 2013,127, S24–S36. 10.1016/j.jenvman.2013.01.014 23422359

[10] Liu Y; Ahiablame LM; Bralts VF; Engel BA Enhancing a rainfall-runoff model to assess the impacts of BMPs and LID practices on storm runoff. J. Environ. Manag. 2015,147,12–23. 10.1016/j.jenvman.2014.09.005 25261748

[11] Gene Y. Using GWLF for development of “Reference Watershed Approach” TMDLs. In Proceedings of the ASAE/CSAE Annual International Meeting, Ottawa, ON, Canada, 1–4 8 2004.

[12] Kang MS; Park SW; Lee JJ; Yoo KH Applying SWAT for TMDL programs to a small watershed containing rice paddy fields. Agric. Water Manag. 2006, 79,72–92.

[13] Corrales J; Naja GM; Bhat MG; Miralles-Wilhelm F. Water quality trading opportunities in two sub-watersheds in the northern Lake Okeechobee watershed. J. Environ. Manag. 2017, 196, 544–559. 10.1016/j.jenvman.2017.03.061 28351821

[14] Hurley SE; Forman RTT Stormwater ponds and biofilters for large urban sites: Modeled arrangements that achieve the phosphorus reduction target for Boston’s Charles River, USA. Ecol. Eng. 2011, 37, 850–863.

[15] Jones KW; Cannon JB; Saavedra FA; Kampf SK; Addington RN; Cheng AS; MacDonald LH; Wilson C; Wolk B. Return on investment from fuel treatments to reduce severe wildfire and erosion in a watershed investment program in Colorado. J. Environ. Manag. 2017,198, 66–77. 10.1016/j.jenvman.2017.05.023 28501609

[16] Imhoff J; Donigian A. History and evolution of watershed modeling derived from the Stanford Watershed Model In Watershed Models; Singh VP, Frevert DK, Eds.; CRC Press: Boca Raton, FL, USA, 2005; pp. 21–45.

[17] Daniel EB; Camp JV; LeBoeuf EJ; Penrod JR; Dobbins JP; Abkowitz MD Watershed modeling and its applications: A state-of-the-art review. Open Hydrol. J. 2011, 5, 26–50.

[18] Crawford NH; Burges SJ History of the Stanford Watershed Model. Water Resour. 2004, 6,1–3.

[19] Bicknell BR; Imhoff JC; Kittle JL Jr.; Donigian AS Jr.; Johanson RC Hydrologic Simulation Program—FORTRAN (HSPF): User’s Manual for Release 10; Report No. EPA/600/R-93/174; U.S. EPA Environmental Research Lab: Athens, Greece, 1993.

[20] Arnold JG; Srinivasan R; Muttiah RS; Williams JR Large-area hydrologic modeling and assessment: Part I. Model development. J. Am. Water Resour. Assoc. 1998, 34, 73–89.

[21] Young RA; Onstad CA; Bosch DD; Anderson WP AGNPS: A non-point-source pollution model for evaluating agricultural watersheds. J. Soil Water Conserv. 1989, 44,168–173.

[22] Lim KJ; Engel BA; Kim Y; Harbor J. Development of the Long-Term Hydrologic Impact Assessment (L-THIA) WWW Systems. In Proceedings of the Sustaining the Global Farm—Selected Papers from the 10th International Soil Conservation Organization Meeting, West Lafayette, IN, USA, 24–29 May 1999; pp. 1018–1023.

[23] Haith DA; Shoemaker LL Generalized watershed loading functions for stream flow nutrients. J. Am. Water Resour. Assoc. 1987, 23, 471–478.

[24] Pitt R. Unique features of the source loading and management model (SLAMM). J. Water Resour. Plan. Manag. 1997, 6,13–37.

[25] Metcalf E; University of Florida and Water Resources Engineers, Inc Storm Water Management Model, Volume I-Final Report; EPA Report 11024 DOC 07/71 (NTIS PB-203289); Environmental Protection Agency: Washington, DC, USA, 1971; p. 352.

[26] Whittemore RC The BASINS model. Water Environ. Technol. 1998,10,57–61.

[27] Ning SK; Chang NB; Jeng KY; Tseng YH Soil erosion and non-point source pollution impacts assessment with the aid of multi-temporal remote sensing images. J. Environ. Manag. 2006, 79, 88–101. 10.1016/j.jenvman.2005.05.019 16182435

[28] Brown ME; Racoviteanu AE; Tarboton DG; Gupta AS; Nigro J; Policelli F; Habib S; Tokay M; Shrestha MS; Bajracharya S; An integrated modeling system for estimating glacier and snow melt driven streamflow from remote sensing and earth system data products in the Himalayas. J. Hydrol. 2014, 519,1859–1869.

[29] Elewa HH; Qaddah AA Groundwater potentiality mapping in the Sinai Peninsula, Egypt, using remote sensing and GIS-watershed-based modeling. Hydrogeol. J. 2011,19, 613–628.

[30] Afan HA; El-shafie A; Mohtar WHMW; Yaseen ZM Past, present and prospect of an Artificial Intelligence (AI) based model for sediment transport prediction. J. Hydrol. 2016, 541, 902–913.

[31] Chiogna G; Marcolini G; Liu W; Perez Ciria T; Tuo Y. Coupling hydrological modeling and support vector regression to model hydropeaking in alpine catchments. Sci. Total Environ. 2018, 633, 220–229. 29573688 10.1016/j.scitotenv.2018.03.162

[32] Lafdani EK; Nia AM; Ahmadi A. Daily suspended sediment load prediction using artificial neural networks and support vector machines. J. Hydrol. 2013, 478, 50–62.

[33] Borah DK; Bera M. Watershed-scale Hydrologic and Nonpoint Source Pollution Models: Review of Mathematical Bases. Trans. ASAE 2003, 46,1553–1566.

[34] Deliman PN; Glick RH; Ruiz CE Review of Watershed Water Quality Models; TR W-99–1; U.S. Army Corps of Engineers: Washington DC, USA, 1999.

[35] Borah DK; Bera M. Watershed-scale Hydrologic and Nonpoint Source Pollution Models: Review of Applications. Trans. ASAE 2004, 47, 789–803.

[36] Fu B; Merritt WS; Croke BFW; Weber TR; Jakeman AJ A review of catchment-scale water quality and erosion models and a synthesis of future prospects. Environ. Model. Softw. 2019,114, 75–97.

[37] Whitehead PG; Wilson E; Butterfield D. A semi-distributed Integrated Nitrogen model for multiple source assessment in Catchments (INCA): Part I—model structure and process equations. Scie. Total Environ. 1998, 210, 547–558.

[38] McMahon G; Roessler C. A regression-based approach to understand baseline total nitrogen loading for TMDL planning. Proc. Water Environ. Fed. 2002, 2002,1277–1303.

[39] Miller S; Semmens D; Goodrich D; Hernandez M; Miller R; Kepner W; Guertin D. The automated geospatial watershed assessment tool. Environ. Model. Softw. 2007,22, 365–377.

[40] Shen J; Parker A; Riverson J. A new approach for a windows-based watershed modeling system based on a database-supporting architecture. Environ. Model. Softw. 2005, 20,1127–1138.

[41] NOAA. Nonpoint-Source Pollution and Erosion Comparison Tool (N-SPECT): Technical Guide; NOAA/CSC/RPT 08–5; The National Oceanic and Atmospheric Administration: Charleston, SC, USA, 2008.

[42] Chen CW; Laura JH; Weintraub HZ Watershed Analysis Risk Management Framework (WARMF): Update One. Topical Report 1005181; Goldstein RA, Ed.; EPRI: Palo Alto, CA, USA, 2001.

[43] SWET. WAM Documentation User Manual; Soil and Water Engineering Technology, Inc.: Gainesville, FL, USA, 2018; Available online: http://www.swet.com/documentation (accessed on 11 January 2020).

[44] AQUAVEO. WMS11.0-The All-in-One Watershed Solution. Available online: https://www.aquaveo.com/software/wms-watershed-modeling-system-introduction (accessed on 12 February 2019).

[45] Brown LC; Barnwell TO The Enhanced Stream Water Quality Models QUAL2E and QUAL2E-UNCAS: Documentation and User Model; Environmental Research Laboratory, Office of Research and Development, US: Washington, DC, USA, 1987.

[46] Wool TA; Ambrose RB; Martin JL; Comer EA; Tech T. Water Quality Analysis Simulation Program (WASP). Available online: https://www.epa.gov/ceam/water-quality-analysis-simulation-program-wasp (accessed on 8 May 2019).

[47] Schoumans O; Mol-Dijkstra J; Akkermans L; Roest C. SIMPLE: Assessment of non-point phosphorus pollution from agricultural land to surface waters by means of a new methodology. Water Sci. Technol. 2002, 45, 177–182. 12079100

[48] Yuan L. Simulation of Soil Erosion and Sediment Yield System in Watershed Based on CA and GIS; Analytical, Institute of Mountain Hazards and Environment, Chinese Academy of Sciences (CAS): Chengdu, China, 2003.

[49] USDA. Urban Hydrology for Small Watersheds; TR-55; United States Department of Agriculture, Natural Resources Conservation Service: Washington, DC, USA, 1986; p. 164.

[50] Lin JP Review of Published Export Coefficient and Event Mean Concentration (EMC) Data; TN-WRAP-04–3; Engineer Research and Development Center: Vicksburg, MS, USA, 2004.

[51] Novotny V. A Review of Hydrologic and Water Quality Models Used for Simulation of Agricultural Pollution In Developments in Environmental Modelling; Elsevier: Amsterdam, The Netherlands, 1986; Volume 10, pp. 9–35.

[52] Engel B; Theller L. Long Term Hydrologic Impact Analysis (L-THIA). Available online: https://engineering.purdue.edu/~{}lthia/ (accessed on 15 February 2019).

[53] Park YS; Lim KJ; Theller L; Engel BA NPSLab. Desktop Models Available online: http://npslab.kongju.ac.kr/#service (accessed on 15 February 2019).

[54] Park YS; Lim KJ; Theller L; Engel BA L-THIA GIS Manual; Purdue University: West Lafayette, IN, USA, 2013.

[55] Li F; Liu Y; Engel BA; Chen J; Sun H. Green infrastructure practices simulation of the impacts of land use on surface runoff: Case study in Ecorse River watershed, Michigan. J. Environ. Manag. 2019,233, 603–611. 10.1016/j.jenvman.2018.12.078 30597354

[56] Zhang J; Shen T; Liu M; Wan Y; Liu J; Li J. Research on non-point source pollution spatial distribution of Qingdao based on L-THIA model. Math. Comput. Model. 2011, 54,1151–1159.

[57] You YY; Jin WB; Xiong QX; Xue L; Ai TC; Li BL Simulation and validation of non-point Source nitrogen and phosphorus loads under different land uses in Sihu basin, Hubei province, China. Procedia Environ. Sci. 2012,13,1781–1797.

[58] Liu Y; Theller LO; Pijanowski BC; Engel BA Optimal selection and placement of green infrastructure to reduce impacts of land use change and climate change on hydrology and water quality: An application to the Trail Creek Watershed, Indiana. Sci. Total Environ. 2016, 553,149–163. 26925727 10.1016/j.scitotenv.2016.02.116

[59] Wright TJ; Liu Y; Carroll NJ; Ahiablame LM; Engel BA Retrofitting LID Practices into Existing Neighborhoods: Is It Worth It? Environ. Manag. 2016, 57, 856–867. 10.1007/s00267-015-0651-5 26725052

[60] Eslinger DL; Carter HJ; Pendleton M; Burkhalter S; Allen M. User’s Manual for OpenNSPECT, Version 1.2.Charleston; National Oceanic and Atmospheric Administration (NOAA) Coastal Services Center: Charleston, NC, USA, 2014.

[61] NOAA. OpenNSPECT. Available online: https://coast.noaa.gov/digitalcoast/tools/opennspect.html (accessed on 3 March 2019).

[62] Middleton T; Libes S. Integrating N-Spect with the Development of a Management Plan for the Kingston Lake Watershed; Coastal Carolina University: Conway, SC, USA, 2007.

[63] Maina J; de Moel H; Vermaat JE; Bruggemann JH; Guillaume MM; Grove CA; Madin JS; Mertz-Kraus R; Zinke J. Linking coral river runoff proxies with climate variability, hydrology and land-use in Madagascar catchments. Mar. Pollut. Bull. 2012, 64, 2047–2059. 22853989 10.1016/j.marpolbul.2012.06.027

[64] Butler JR; Wong GY; Metcalfe DJ; Honzak M; Pert PL; Rao N; Van Grieken ME; Lawson T; Bruce C; Kroon FJ An analysis of trade-offs between multiple ecosystem services and stakeholders linked to land use and water quality management in the Great Barrier Reef, Australia. Agric. Ecosyst. Environ. 2013, 180,176–191.

[65] Tulloch VJD; Brown CJ; Possingham HP; Jupiter SD; Maina JM; Klein C. Improving conservation outcomes for coral reefs affected by future oil palm development in Papua New Guinea. Biol. Conserv. 2016, 203, 43–54.

[66] Alvarez-Romero JG; Wilkinson SN; Pressey RL; Ban NC; Kool J; Brodie J. Modeling catchment nutrients and sediment loads to inform regional management of water quality in coastal-marine ecosystems: A comparison of two approaches. J. Environ. Manag. 2014,146,164–178. 10.1016/j.jenvman.2014.07.007 25173725

[67] Burkhalter S; Pendleton M. OpenSPECT: An Open Source Version of the Nonpoint Source Pollution and Erosion Comparison Tool. Available online: https://slideplayer.com/slide/4557881/ (accessed on 19 February 2019).

[68] Wu RS; Lin IW Modification of generalized watershed loading functions (GWLF) for daily flow simulation. Paddy Water Environ. 2015,13, 269–279.

[69] Haith DA; Mandel R; Wu RS GWLF: Generalized Watershed Loading Functions Version 2.0 User’s Manual; Cornell University: Ithaca, NY, USA, 1992.

[70] Evans BM MapShed GIS-Based Watershed Modeling Tool. Available online: https://wikiwatershed.org/help/model-help/mapshed/ (accessed on 2 January 2020).

[71] Rossman LA Storm Water Management Model User’s Manual Version 5.1; EPA/600/R-14/413b; U.S. Environmental Protection Agency: Cincinnati, OH, USA, 2015.

[72] Hydrologic Engineering Center Storage, Treatment, Overflow, Runoff Model “STORM”: Users Manual; University of California: Oakland, CA, USA, 1977.

[73] Schneiderman EM; Pierson DC; Lounsbury DG; Zion MS Modeling the hydrochemistry of the Cannonsville watershed with Generalized Watershed Loading Functions (GWLF). J. Am. Water Resour. Associ. 2002, 38,1323–1347.

[74] Georgas N; Rangarajan S; Farley KJ; Jagupilla SCK AVGWLF-based estimation of nonpoint source nitrogen loads generated within long island sound subwatersheds. J. Am. Water Resour. Associ. 2009, 45, 715–733.

[75] Elliott A; Trowsdale S. A review of models for low impact urban stormwater drainage. Environ. Model. Softw. 2007,22, 394–405.

[76] Qi Z; Kang G; Chu C; Qiu Y; Xu Z; Wang Y. Comparison of SWAT and GWLF model simulation performance in humid south and semi-arid north of China. Water 2017, 9, 567.

[77] Loading Simulation Program in C++ (LSPC) Version 3.1 User’s Manual; Tetra Tech: Fairfax, VA, USA, 2009.

[78] EPA US LSPC-Loading Simulation Program C. Available online: https://github.com/USEPA/LSPC-Loading-Simulation-Program (accessed on 10 March 2019).

[79] Tech Tetra. Loading Simulation Program in C++ (LSPC) Version 5.0 User’s Manual; EPA Contract # EP-R8–12-04; Tetra Tech: Cleveland, OH, USA, 2017.

[80] Sharma S; Srivastava P; Fang X; Kalin L. Performance comparison of Adoptive Neuro-Fuzzy Inference System (ANFIS) with Loading Simulation Program C++ (LSPC) model for streamflow simulation in El Niño Southern Oscillation (ENSO)-affected watershed. Expert Syst. Appl. 2015, 42, 2213–2223.

[81] Shen J; Zhao Y. Combined Bayesian statistics and load duration curve method for bacteria nonpoint source loading estimation. Water Res. 2010, 44, 77–84. 19781737 10.1016/j.watres.2009.09.002

[82] Huang JJ; Xiang W. Investigation of point source and non-point source pollution for Panjiakou Reservoir in North China by modelling approach. Water Qual. Res. J. Can. 2015, 50,167–181.

[83] WinSLAMM Version 10.4.1. Available online: http://winslamm.com/ (accessed on 6 April 2019).

[84] Pitt R. WinSLAMM v 10.2 User’s Guide. Available online: http://winslamm.com/ (accessed on 5 May 2019).

[85] Pitt R; Voorhees J. SLAMM, the Source Loading and Management Model; CRC Press: Boca Raton, FL, USA, 2002; pp. 103–109.

[86] Myllyoja R; Baroudi H; Pitt R; Paluzzi J. Use of SLAMM in evaluating best management practices. Models Appl. Urban Water Syst. Monogr. 2001, 9,131–141.

[87] Selbig W; Fienen M; Horwatich J; Bannerman R. The effect of particle size distribution on the design of urban stormwater control measures. Water 2016, 8,17.

[88] Pitt R. Small Storm Flow and Particulate Washoff Contributions to Outfall Discharges; University of Wisconsin-Madison: Madison, WI, USA, 1987.

[89] Systech Water Resources: WARMF Software. Available online: http://warmf.com/home/ (accessed on 8 April 2019).

[90] Herr JW; Chen CW WARMF: Model Use, Calibration, and Validation. Trans. ASABE 2012, 55,1385–1394.

[91] Dayyani S; Prasher SO; Madani A; Madramootoo CA Development of DRAIN-WARMF model to simulate flow and nitrogen transport in a tile-drained agricultural watershed in Eastern Canada. Agric. Water Manag. 2010, 98,55–68.

[92] Geza M; Poeter EP; McCray JE Quantifying predictive uncertainty for a mountain-watershed model. J. Hydrol. 2009, 376,170–181.

[93] McCray JE Software Review: Watershed Analysis Risk Management Framework (WARMF). Southwest Hydrol. 2006, 5, 41.

[94] Liu L. Application of a Hydrodynamic and Water Quality Model for Inland Surface Water Systems. 2018 Available online: https://www.intechopen.com/books/applications-in-’water-systems-management-and-modeling/application-of-a-hydrodynamic-and-water-quality-model-for-inland-surface-water-systems (accessed on 11 January 2020).

[95] Abbaspour K; Vaghefi S; Srinivasan R. A Guideline for Successful Calibration and Uncertainty Analysis for Soil and Water Assessment: A Review of Papers from the 2016 International SWAT Conference. Water 2018,10, 6.

[96] Moriasi D; Wilson BN; Douglas-Mankin K; Arnold J; Gowda P. Hydrologic and water quality models: Use, calibration, and validation. Trans. ASABE 2012, 55,1241–1247.

[97] Young RA; Onstad C; Bosch D; Singh V. An Agricultural Nonpoint Source Model. In Proceedings of the Workshop on Computer Applications in Water Management, Fort Collins, CO, USA, 23–25 May 1995.

[98] Theurer FD; Bingner R. Fact Sheet: Pollutant Loading Modeling Environment—AGNPS. Available online: https://www.wcc.nrcs.usda.gov/ftpref/wntsc/HfeH/AGNPS/downloads (accessed on 02 May 2019).

[99] USDA. AGricultural Non-Point Source Pollution Model. Available online: http://go.usa.gov/KFO (accessed on 15 April 2019).

[100] Bosch D; Theurer F; Bingner R; Felton G; Chaubey I. Evaluation of the AnnAGNPS water quality model. In Proceedings of the 1988 ASAE Annual International Meeting, Orlando, FL, USA, 11–16 July 1998; pp. 45–54.

[101] Theurer FD; Bingner R. Fact Sheet: Watershed-Scale Pollutant Loading Model-AnnAGNPS v5.5; USDA: Oxford, MS, USA, 2019.

[102] Penman HL Natural evaporation from open water, bare soil and grass. Proc. R. Soc. Lond. Ser. A Math. Phys. Sci. 1948,193,120–145. 18865817 10.1098/rspa.1948.0037

[103] Renard KG; Foster GR; Weesies GA; McCool DK; Yoder DC Predicting Soil Erosion By Water: A Guide to Conservation Planning with the Revised Universal Soil Loss Equation (RUSLE); USDA: Washington, DC, USA, 1996; p. 703.

[104] Bingner RL; Theurer FD; Yuan Y. AnnAGNPS Technical Processes Documentation; USDA: Washington, DC, USA, 2018.

[105] Li Z; Luo C; Xi Q; Li H; Pan J; Zhou Q; Xiong Z. Assessment of the AnnAGNPS model in simulating runoff and nutrients in a typical small watershed in the Taihu Lake basin, China. Catena 2015,133, 349–361.

[106] Karki R; Tagert MLM; Paz JO; Bingner RL Application of AnnAGNPS to model an agricultural watershed in East-Central Mississippi for the evaluation of an on-farm water storage (OFWS) system. Agric. Water Manag. 2017,192,103–114.

[107] Chahor Y; Casalí J; Giménez R; Bingner RL; Campo MA; Goñi M. Evaluation of the AnnAGNPS model for predicting runoff and sediment yield in a small Mediterranean agricultural watershed in Navarre (Spain). Agric. Water Manag. 2014,134,24–37.

[108] USDA-ARS. SWAT Soil & Water Assessment Tool. Available online: https://swat.tamu.edu/ (accessed on 18 April 2019).

[109] Arnold JG; Moriasi DN; Gassman PW; Abbaspour KC; White MJ; Srinivasan R; Santhi C; Harmel R; Van Griensven A; Van Liew MW SWAT: Model use, calibration, and validation. Trans. ASABE 2012, 55, 1491–1508.

[110] Abbaspour KC; Rouholahnejad E; Vaghefi S; Srinivasan R; Yang H; Klove B. A continental-scale hydrology and water quality model for Europe: Calibration and uncertainty of a high-resolution large-scale SWAT model. J. Hydrol. 2015, 524, 733–752.

[111] Gassman PW; Reyes MR; Green CH; Arnold JG The soil and water assessment tool: Historical development, applications, and future research directions. Trans. ASABE 2007, 50,1211–1250.

[112] Williams J; Berndt H. Sediment Yield Prediction Based on Watershed Hydrology; American Society of Agricultural Engineering: St. Joseph, MI, USA, 1976.

[113] Neitsch SL; Arnold JG; Kiniry JR; Williams JR Soil and water Assessment Tool Theoretical Documentation Version 2009; Texas Water Resources Institute: College Station, TX, USA, 2011.

[114] Stehr A; Debels P; Romero F; Alcayaga H. Hydrological modeling with SWAT under conditions of limited data availability: Evaluation of results from a Chilean case study. Hydrol. Sci. J. 2010,53, 588–601.

[115] SWAT Literature Database for Peer-Reviewed Journal Articles. Available online: https://www.card.iastate.edu/swat_articles/ (accessed on 27 February 2019).

[116] Google Group: ArcSWAT. Available online: https://groups.google.com/forum/#!forum/arcswat (accessed on 27 February 2019).

[117] Yuan L; Forshay KJ Using SWAT to evaluate streamflow and lake sediment loading in the Xinjiang River Basin with limited data. Water 2020,12, 39. 10.3390/w12010039 PMC7513863 32983578

[118] SWAT-CUP. Available online: https://swat.tamu.edu/software/swat-cup/ (accessed on 30 April 2019).

[119] Zhang D; Chen X; Yao H; Lin B. Improved calibration scheme of SWAT by separating wet and dry seasons. Ecol. Model. 2015, 301,54–61.

[120] Muleta MK Improving Model Performance Using Season-Based Evaluation. J. Hydrol. Eng. 2012, 17, 191–200.

[121] Gao X; Chen X; Biggs T; Yao H. Separating wet and dry years to improve calibration of SWAT in Barrett Watershed, Southern California. Water 2018,10, 274.

[122] U.S. EPA: Storm Water Management Model (SWMM). Available online: https://www.epa.gov/water-research/storm-water-management-model-swmm (accessed on 22 March 2019).

[123] Gironás J; Roesner LA; Davis J; Rossman LA; Supply W. Storm Water Management Model Applications Manual; EPA/600/R-09/000; National Risk Management Research Laboratory: Cincinnati, OH, USA, 7 2009.

[124] Obropta CC; Kardos JS Review of Urban Stormwater Quality Models: Deterministic, Stochastic, and Hybrid Approaches1. JAWRA J. Am. Water Resour. Associ. 2007, 43,1508–1523.

[125] McGarity AE Watershed Systems Analysis for Urban Storm-Water Management to Achieve Water Quality Goals. J. Water Resour. Plan. Manag. 2013,139, 464–477.

[126] Zhang K; Chui TFM; Yang Y. Simulating the hydrological performance of low impact development in shallow groundwater via a modified SWMM. J. Hydrol. 2018, 566, 313–331.

[127] Niazi M; Nietch C; Maghrebi M; Jackson N; Bennett BR; Tryby M; Massoudieh A. Storm Water Management Model: Performance Review and Gap Analysis. J. Sustain. Water Built Environ. 2017, 3, 04017002. 10.1061/jswbay.0000817 PMC7326159 32607450

[128] Jang S; Cho M; Yoon J; Yoon Y; Kim S; Kim G; Kim L; Aksoy H. Using SWMM as a tool for hydrologic impact assessment. Desalination 2007, 212, 344–356.

[129] U.S. EPA: BASINS Download and Installation. Available online: https://www.epa.gov/ceam/basins-download-and-installation (accessed on 22 March 2019).

[130] USGS: Hydrological Simulation Program—Fortran. Available online: https://water.usgs.gov/software/HSPF/ (accessed on 5 May 2019).

[131] Duda PB; Hummel PR; Donigian ASJ; Imhoff JC BASIN/HSPF: Model use, Calibration, and Validation. Trans. ASABE 2012, 55,1523–1547.

[132] Skahill BE Use of the Hydrological Simulation Program—FORTRAN (HSPF) Model for Watershed Studies; ERDC/TN SMART-04–1; Army Engineer Research and Development Center: Vicksburg, MS, USA, 2004.

[133] Bicknell BR; Imhoff JC; Kittle JL Jr.; Donigian AS Jr.; Johanson RC Hydrological Simulation Program—FORTRAN User’s Manual for Version 11; Environmental Protection Agency Report No. EPA/600/R-97/080; US Environmental Protection Agency: Athens, Greece, 1997.

[134] Saleh A; Du B. Evaluation of SWAT and HSPF Within BASINS Program For The Upper North Bosque River Watershed in Central Texas. Trans. ASAE 2004, 47,1039–1049.

[135] AQUA TERRA: Bibliography for HSPF and Related References. Available online: http://www.aquaterra.com/resources/hspfsupport/hspfbib.php (accessed on 22 February 2019).

[136] Huo SC; Lo SL; Chiu CH; Chiueh PT; Yang CS Assessing a fuzzy model and HSPF to supplement rainfall data for nonpoint source water quality in the Feitsui reservoir watershed. Environ. Model. Softw. 2015, 72,110–116.

[137] Xie H; Lian Y. Uncertainty-based evaluation and comparison of SWAT and HSPF applications to the Illinois River Basin. J. Hydrol. 2013, 481,119–131.

[138] Im S; Brannan K; Mostaghimi S; Cho J. A Comparison of SWAT and HSPF Models for Simulating Hydrologic and Water Quality Responses from an Urbanizing Watershed. In Proceedings of the 2003 ASAE Annual International Meeting, Las Vegas, NV, USA, 27–30 July 2003.

[139] SWET. WAM Toolbar for ArcMAP 10.4.1. Available online: http://www.swet.com/wam-for-arcmap-100/ (accessed on 10 May 2019).

[140] Bottcher AB; Whiteley BJ; James AI; Hiscock JGA Watershed Assessment Model (WAM) Applications in Florida. In Proceedings of the 2012 Esri International User Conference (ESRI), San Diego, CA, USA, 23–27 July 2012; pp. 1–17.

[141] SWET. Final Report: Watershed Assessment Model (WAM): Calibration and Uncertainty and Sensitivity Analyses; Soil and Water Engineering Technology, Inc.: Gainesville, FL, USA, 2015.

[142] Bottcher D. Watershed Assessment Model (WAM) Evaluation of the Suwannee River Basin; USGS Open-File Report 2004–1332 Paperback; BiblioGov: Cedar Key, FL, USA, 2004.

[143] U.S. EPA: Automated Geospatial Watershed Assessment (AGWA) Tool. Available online: https://www.epa.gov/water-research/automated-geospatial-watershed-assessment-agwa-tool (accessed on 4 April 2019).

[144] Welcome to the Automated Geospatial Watershed Assessment Tool. Available online: https://www.tucson.ars.ag.gov/agwa/ (accessed on 22 February 2019).

[145] Goodrich DC; Guertin DP; Burns IS; Nearing MA; Stone JJ; Wei H; Heilman P; Hernandez M; Spaeth K; Pierson F; AGWA: The Automated Geospatial Watershed Assessment Tool to Inform Rangeland Management. Rangelands 2011, 33, 41–47.

[146] Yang B; Li M-H Assessing planning approaches by watershed streamflow modeling: Case study of The Woodlands; Texas. Landsc. Urban Plan. 2011, 99, 9–22.

[147] USDA-ARS; U.S. EPA; Wyoming, U.O. AGWA 3.x User Guide; U.S. EPA: Tucson, AZ, USA, 2017.

[148] EPA, U.S. BASINS 4.1 (Better Assessment Science Integrating point & Non-point Sources) Modeling Framework; National Exposure Research Laboratory: RTP, NC, USA, 2015.

[149] Crossette E; Panunto M; Kuan C; Mohamoud YM Application of BASINS/HSPF to Data-scarce Watersheds; EPA/600/R-15/007; U.S. EPA: Washington, DC, USA, 2015.

[150] Zhou Z; Ouyang Y; Li Y; Qiu Z; Moran M. Estimating impact of rainfall change on hydrological processes in Jianfengling rainforest watershed, China using BASINS-HSPF-CAT modeling system. Ecol. Eng. 2017,105,87–94.

[151] AQUAVEO. WMS User Manual v10.1; Aquaveo: Provo, UT, USA, 2012.

[152] Erturk A; Gurel M; Baloch MA; Dikerler T; Varol E; Akbulut N; Tanik A Application of watershed modeling system (WMS) for integrated management of a watershed in Turkey. J. Environ. Sci. Health A Tox Hazard Subst. Environ. Eng. 2006, 41, 2045–2056. 16849145 10.1080/10934520600780693

[153] Soussa H; El Feel AA; Alfy SZ; Yousif MSM Flood hazard in Wadi Rahbaa area, Egypt. Arab. J. Geosci. 2010,5,45–52.

[154] Engel B; Storm D; White M; Arnold J; Arabi M. A Hydrologic/Water Quality Model Application Protocol. J. Am. Water Resour. Associ. 2007,43,1223–1236.

[155] Yuan L. Study on Spatio-Temporal Changes Process and Mechanism of Precipitation, Runoff and Soil Erosion in the Poyang Lake Basin during the past 50 Years; Institution of Geography & Limnology, Chinese Academy of Sciences (CAS): Nanjing, China, 2011.

[156] Baffaut C; Dabney SM; Smolen MD; Youssef MA; Bonta JV; Chu ML; Guzman JA; Shedekar VS; Jha MK; Arnold JG Hydrologic and Water Quality Modeling: Spatial and Temporal Considerations. Trans. ASABE 2015, 58,1661–1680.

[157] Bingner RL; Theurer FD; Cronshey RG; Darden RW AnnAGNPS Technical Processes. 2009 Available online: http://www.ars.usda.gov/Research/docs.htm?docid=5199 (accessed on 12 February 2019).

[158] Moriasi DN; Arnold JG; Van Liew MW; Bingner RL; Harmel RD; Veith TL Model evaluation guidelines for systematic quantification of accuracy in watershed simulations. Trans. ASABE 2007, 50, 885–900.

[159] Daggupati P; Pai N; Ale S; Douglas-Mankin KR; Zeckoski RW; Jeong J; Parajuli PB; Saraswat D; Youssef MA A Recommended Calibration and Validation Strategy for Hydrologic and Water Quality Models. Trans. ASABE 2015, 58,1705–1719.

[160] Lim KJ; Engel BA; Tang Z; Muthukrishnan S; Choi J; Kim K. Effects of calibration on L-THIA GIS runoff and pollutant estimation. J. Environ. Manag. 2006, 78,35–43. 10.1016/j.jenvman.2005.03.014 16112801

[161] Eckhardta K; Arnold JG Automatic calibration of a distributed catchment model. J. Hydrol. 2001, 251, 103–109.

[162] Abbaspour KC SWAT-CUP: SWAT Calibration and Uncertainty Programs—A User Manual; Swiss Federal Insitute of Aquatic Sciences and Technology: Zurich, Switzerland, 2015.

[163] Yang J; Reichert P; Abbaspour KC; Xia J; Yang H. Comparing uncertainty analysis techniques for a SWAT application to the Chaohe Basin in China. J. Hydrol. 2008, 358,1–23.

[164] Abbaspour K; Vejdani M; Haghighat S; Yang J. SWAT-CUP calibration and uncertainty programs for SWAT. In Proceedings of the MODSIM 2007 International Congress on Modelling and Simulation, Modelling and Simulation Society of Australia and New Zealand, Christchurch, New Zealand, 10–13 December 2007; pp. 1596–1602.

[165] Liao Q; Shen Z. Uncertainties in agricultural nonpoint source pollution simulation: Research progress. Chin. J. Ecol. 2011, 30,1542–1550.

[166] Muleta MK; Nicklow JW Sensitivity and uncertainty analysis coupled with automatic calibration for a distributed watershed model. J. Hydrol. 2005, 306,127–145.

[167] EPA US; Sinshaw T; Yuan L; Forshay KJ A Review of Watershed and Water Quality Tools for Nutrient Fate and Transport; EPA/600/R-19/232; Center for Environmental Solutions & Emergency Response | Groundwater Characterization & Remediation Division, Office of Research and Development (EPA): Ada, OK, USA, 12 2019.

